# O-GlcNAcylation of UGDH regulates its activity and remodels the extracellular matrix to facilitate tumor growth

**DOI:** 10.1038/s41418-025-01591-8

**Published:** 2025-10-06

**Authors:** Bingyi Lin, Junjie Zhou, Didi Geng, Siyuan Chai, Xuanming Zhang, Zengle Zhang, Jiating Hu, Qin Tang, Xiaoming Chen, Wen Yi, Liming Wu

**Affiliations:** 1 Department of Hepatobiliary and Pancreatic Surgery, The First Affiliated Hospital, Zhejiang University, School of Medicine, Hangzhou, China; 2 Department of Biochemistry, College of Life Sciences, Zhejiang University, Hangzhou, China; 3 Department of General Surgery, Center for Metabolism Research, The Fourth Affiliated Hospital of Zhejiang University School of Medicine and International School of Medicine, International Institutes of Medicine, Zhejiang University, Yiwu, China; 4 School of Laboratory Medicine and Life Sciences, Wenzhou Medical University, Wenzhou, China

**Keywords:** Cancer microenvironment, Cancer metabolism, Glycobiology, Glycoconjugates

## Abstract

The tumor microenvironment is an immunosuppressive niche that contributes to tumor growth by downregulating immune cell functions or restraining immune cell infiltration. The underlying mechanisms are not still poorly understood. Here, we demonstrate that O-linked N-acetylglucosamine (O-GlcNAcylation), a prevalent form of protein glycosylation, contributes to establishing the immunosuppressive niche through regulating the metabolic and non-metabolic functions of uridine diphosphate glucose dehydrogenase (UGDH). Tumor cells carrying O-GlcNAcylation-deficient UGDH showed reduced xenograft tumor growth and improved survival in mice. Cytometry by time-of-flight (CyTOF) analysis suggests UGDH O-GlcNAcylation negatively correlates with cytotoxic CD8^+^ T cell infiltration. O-GlcNAcylation on serine 350 of UGDH is located within the UDP-binding domain, and the subsequent extensive all-atom molecular dynamics simulations reveal that O-GlcNAcylation reinforces hydrogen-bonding interaction and enzymatic activity of UGDH, leading to enhanced hyaluronic acid (HA) synthesis in the extracellular matrix. Moreover, O-GlcNAcylation of UGDH reduces CD8^+^ T cell infiltration by decreasing the chemokine CXCL10 expression. Specifically, O-GlcNAcylation enhances UGDH interaction with KPNA2 to compete with STAT1, and suppresses translocation of STAT1 into the nucleus, thereby transcriptionally downregulating CXCL10 expression. Thus, our study identifies UGDH O-GlcNAcylation as a key regulator of tumor immunity and further suggests a potential strategy for enhancing immunotherapy.

## Introduction

The latest update on cancer statistics worldwide provided close to 20 million new diagnoses of cancer, and alongside 9.7 million deaths from cancer in one year [1]. The estimates suggest that approximately one in five men or women develops cancer in a lifetime, whereas around one in ten people dies from it. Hence, further understanding the molecular underpinnings of cancer to improve patients’ survival outcomes remains crucial. Recently, protein O-GlcNAcylation has emerged as a critical mechanism regulating cancer development and progression and attracting wide attention [2]. O-GlcNAcylation is a highly dynamic and reversible post-translational modification that occurs on serine/threonine residues of intracellular proteins. It is regulated by O-GlcNAc transferase (OGT) and hydrolase (OGA), which add and remove the GlcNAc moiety, respectively, thereby maintaining the homeostatic level of O-GlcNAcylation in cells. Reprogramming of glucose metabolism leads to abnormal activation of the hexosamine biosynthetic pathway (HBP), which provides the nucleotide sugar donor (UDP-GlcNAc) for O-GlcNAcylation, resulting in hyper-O-GlcNAcylation in tumors [3–5]. Accumulating evidence has revealed the pivotal role of O-GlcNAcylation in regulating tumor initiation, growth, metastasis, and immune response. For example, glucose metabolism promoted O-GlcNAcylation of the lysosome-encapsulated protease Cathepsin B at serine 210, mediated by lysosome-localized O-GlcNAc transferase (OGT), elevating mature Cathepsin B in macrophages and its secretion in the tumor microenvironment (TME), thereby promoting cancer metastasis and chemoresistance [6]. In triple-negative breast cancers, O-GlcNAcylation of Foxp3 and STAT3 inhibited the differentiation of Treg cells into inflammatory Th17 cells, leading to the generation of a tumor immunosuppressor microenvironment [7]. In addition, OGT in exosomes derived from esophageal cancer stem cells (ECSCs) could protect ECSCs from the killing effect of CD8^+^ T cells by upregulating PD-1 expression [8]. Recently, we also reported that O-GlcNAcylation of HGS suppressed the degradation of EGFR and PD-L1 to promote hepatocellular carcinoma development [9, 10]. Consequently, increased O-GlcNAcylation is being considered as a common feature of cancers [11], understanding of which is expected to bring new breakthroughs in cancer biology.

Previously, we developed a mass spectrometry-based strategy for quantitative profiling of O-GlcNAcylated proteins in liver cancer cells using a photocleavable probe [12] and identified UGDH with increased O-GlcNAcylation level. UGDH is the key enzyme in the uronic acid pathway that catalyzes the conversion of UDP-glucose (UDP-Glc) to UDP-glucuronic acid (UDP-GlcUA) [13]. Polymerization of UDP-Glc and UDP-GlcNAc forms hyaluronic acid (HA), an important component of the extracellular matrix (ECM). Based on the Cancer Genome Atlas (TCGA) database analysis, UGDH expression was upregulated in multiple cancer types and positively correlated with poor prognosis. HA is not only involved in maintaining the structural balance of ECM, but also involved in ECM functions such as signal transduction, immune response, and cell proliferation. Besides, HA is also considered a common factor for inducing the formation of immunosuppressed macrophages and interacting with several tumor-promoting cell receptors, including CD44 [14], TLR4 [15], and HABP [16]. Martinez-Ordonez et al. demonstrated HA degradation with a clinical dose of hyaluronidase impaired colorectal tumorigenesis and liver metastasis in vivo, and enabled efficient immune checkpoint blockade therapy by promoting the recruitment of B and CD8^+^ T cells [17]. Thus, UGDH is an oncoprotein associated with tumor immune evasion, but the underlying molecular mechanism remains unclear.

Here, we report a mechanism by which O-GlcNAcylation contributes to suppressing CD8^+^ T cell infiltration to regulate tumor immune evasion and tumor progression. O-GlcNAcylation increased HA generation and reinforced the ECM structure by enhancing UGDH enzyme activity. Moreover, O-GlcNAcylation increased UGDH interaction with KPNA2 to compete with STAT1, which decreased STAT1 translocation to the nucleus to suppress CXCL10 expression. HA accumulation and CXCL10 downregulation work synergistically to diminish CD8^+^ T cell infiltration and promote tumor metastasis in vitro and in vivo. Collectively, these findings not only shed light on the regulatory mechanism governing UGDH biological function but also provide a potential strategy for improved cancer immunotherapy.

## Results

### UGDH correlates with poor prognosis and possesses hyper-O-GlcNAcylation in the tumor

**Fig. 1:**
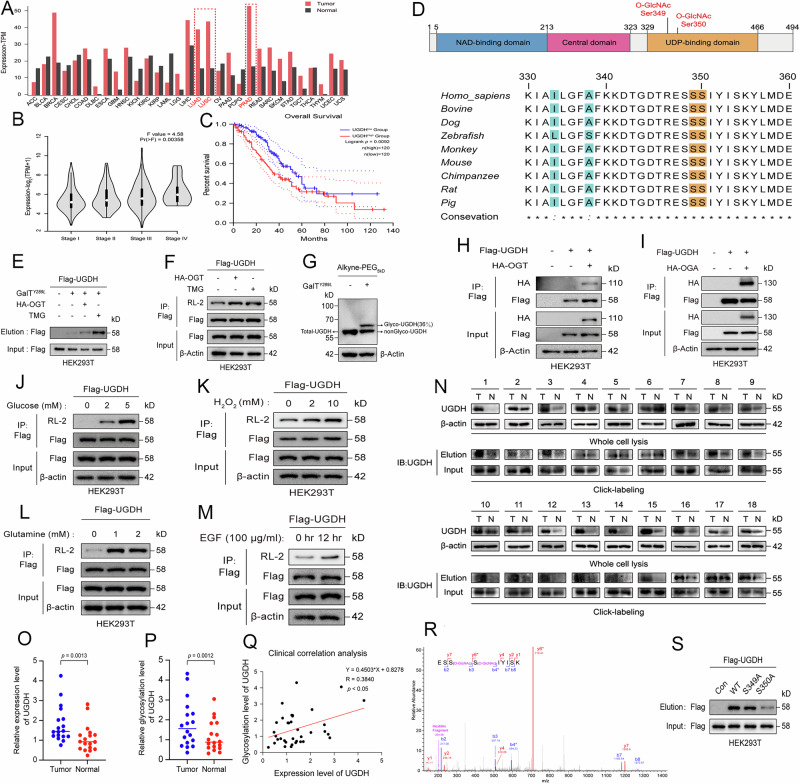
UGDH correlates with poor prognosis and possesses high O-GlcNAcylation in the tumor. **A** TCGA analysis shows that UGDH expression is significantly upregulated in multiple cancer tissues, especially in hepatocellular carcinoma, breast invasive carcinoma, and NSCLC. **B** UGDH expression in NSCLC is higher in patients with stage III-IV than stage I-II according to the Barcelona criteria. **C** Kaplan–Meier analysis demonstrates that high levels of UGDH expression are associated with shorter survival outcomes in NSCLC. **D** UGDH has an evolutionarily conserved amino acid sequence across different species, which comprises residues of potential O-GlcNAcylation. **E**, **F** Analysis of UGDH glycosylation in HEK293T cells overexpressing Flag-tagged UGDH under co-transfection of HA-OGT or TMG treatment. **G** Immunoblotting analysis of the glycosylation ratio of UGDH using enzymatic labeling coupled with alkyne PEG-5kD. **H**, **I** The co-immunoprecipitation of OGT/OGA with UGDH. Immunoblotting analysis of UGDH glycosylation after different nutrient stimuli, including glucose (**J**), H_2_O_2_ (**K**), glutamine (**L**) and EGF (**M**). **N** Immunoblotting analysis of UGDH expression and O-GlcNAcylation levels from primary NSCLC tissues and the matched adjacent tissues. Immunoblotting quantification of UGDH expression (**O**) and O-GlcNAcylation levels (**P**). **Q** Pearson’s correlation test was used to analyze the relationship between the levels of UGDH expression and UGDH O-GlcNAcylation. **R** Identification of O-GlcNAcylation sites on UGDH by high-resolution tandem mass spectrometry spectrum analysis. **S** Verifying the UGDH glycosylation sites using alanine mutations. Results are representative of three biological replicates.

The correlation of UGDH mRNA expression with prognosis in cancers was first analyzed using the GEPIA based on the RNA-seq datasets derived from the TCGA database [18]. Compared with para-cancer tissues, UGDH expression was significantly higher in multiple cancer tissues, especially in hepatocellular carcinoma, breast invasive carcinoma, and non-small-cell lung carcinoma (NSCLC) (Fig. 1A). Further analysis in NSCLC showed that NSCLC patients with III-IV stage displayed higher UGDH expression compared to I-II stage according to the Barcelona criteria (Fig. 1B). The Kaplan–Meier analysis also demonstrated that high levels of UGDH expression significantly correlated with poor prognosis in NSCLC (Fig. 1C).

We next investigated the underlying mechanisms by which UGDH contributes to tumor progression. As UGDH was identified as a potential substrate of O-GlcNAcylation in our glycoproteomic study (Fig. S1A), we first analyzed whether UGDH possesses O-GlcNAcylation. The amino acid sequence of UGDH contains an evolutionarily conserved amino acid sequence across different species, which shows two serine residues for possible O-GlcNAcylation as predicted by the Yin-o-Yang analytical tool (Fig. 1D). We next experimentally confirmed the presence of O-GlcNAcylation using a well-established chemoenzymatic labeling method [19]. The results clearly showed a clear immunoblotting signal in the labeled samples but were absent in the control samples in which the OGT was omitted (Fig. 1E). Expectedly, OGT overexpression or treatment with the OGA inhibitor Thiamet-G (TMG) apparently increased the O-GlcNAcylation signal (Fig. 1F). When ectopically expressed, Flag-tagged UGDH was immunoprecipitated, and it could also be blotted with a pan antibody specific for O-GlcNAcylation. The O-GlcNAcylation ratio of UGDH was approximately 36% (Fig. 1G). We also detected the association of OGT/OGA with UGDH by co-immunoprecipitation (CO-IP) (Fig. 1H, I). Additionally, consistent with its role as a sensor, the O-GlcNAcylation level of UGDH was dynamically responsive to different environmental cues in cell cultures (Fig. 1J–M).

Furthermore, a cohort of 18 pairs of tumoral and adjacent peritumoral tissues from NSCLC patients was collected for analysis. The protein level and O-GlcNAcylation level of UGDH were generally higher in tumoral tissues compared to the peritumoral tissues (Fig. 1N–P). Quantification showed a positive correlation between UGDH protein levels and O-GlcNAcylation levels (Fig. 1Q), suggesting that UGDH O-GlcNAcylation is potentially involved in the malignancy of NSCLC.

To further identify the sites of O-GlcNAcylation on UGDH, we ectopically expressed and purified Flag-tagged UGDH in HEK293T, and subjected the protein to in-gel trypsin digestion, followed by liquid chromatography coupled mass spectrometry (LC-MS) analysis (Fig. 1R). The result identified two putative sites (Ser349 and Ser350) in the evolutionarily conserved sequence, which were then mutated to alanine to generate singlet or doublet mutants as surrogates for glycosylation deficiency. Immunoblotting analysis showed that the glycosylation level of UGDH was largely reduced in the Ser350 mutant, but not the Ser349 mutant, in a panel of tumor cell lines (Fig. S1B–E). Although residual glycosylation level was still detected in the mutant, the result suggests that Ser350 is the major glycosylation site in UGDH.

### UGDH O-GlcNAcylation promotes tumor growth and metastasis in vitro and vivo

**Fig. 2:**
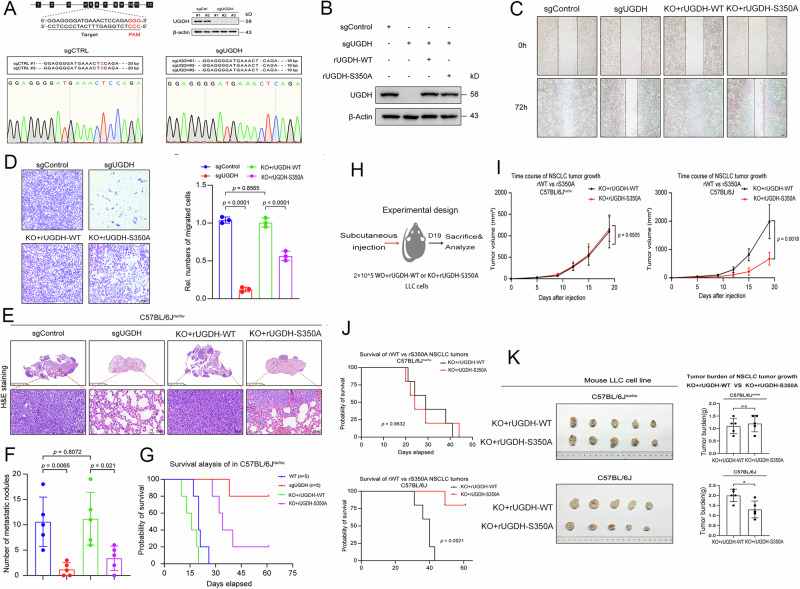
UGDH O-GlcNAcylation promotes tumor growth and metastasis in vitro and vivo. **A** Verification of UGDH deletion in the human A549 cell line. Two sgRNAs targeting UGDH fragments were amplified by PCR and cloned into the pMD™19-T vector for sequencing. Indels are marked in red. Three consecutive bases marked in red indicate the predicted Cas9 cleavage site. **B** A549 cells were depleted of endogenous UGDH by CRISPR-Cas9 and rescued with UGDH-WT or UGDH-S350A. **C** Representative images of the scratch assay at the indicated time performed in A549 cells rescued with UGDH-WT and UGDH-S350A are shown. **D** The transwell assay was used to quantitatively detect the migration ability of genetically modified A549 cells mentioned above. **E**–**G** A549 cells rescued with UGDH-WT or UGDH-S350A were implanted into C57BL/6Jnu/nu mice by tail vein injection to detect cancer cell metastasis ability in vivo. Representative images of H&E-stained sections in dissected lungs 21 days after injection are shown (**E**). The metastatic lesions are quantified based on the staining results of lung sections, the mean ± SEM of metastatic nodules in each of the 5 mice in each group (**F**). The overall survival (OS) of C57Bl/6Jnu/nu mice bearing lung metastases seeded with A549 cells with or without UGDH. *n* = 5 mice per group (**G**). **H** Schematic diagram of subcutaneous injection of LLC UGDH-WT and LLC UGDH-S350A cells and detection of primary tumor growth after 19 days. **I** Tumor growth curves of resultant UGDH-WT and UGDH-350A NSCLC tumors in C57Bl/6Jnu/nu or C57BL/6J mice. *n* = 5 mice per group. Statistics by student’s *t*-test. Tumor volumes were measured three times per week and calculated using the formula V = π/4 × length × width^2^. **J** Overall survival (OS) of C57Bl/6Jnu/nu or C57BL/6J mice bearing a primary tumor seeded with UGDH-WT or UGDH-350A cells. *n* = 5 mice per group. Statistics by Kaplan–Meier test. **K** Tumor graphs and weights are shown for NSCLC UGDH-WT and for UGDH-S350A growing in C57BL/6Jnu/nu or C57BL/6J mice at day 19 after s.c. injection. *N* = 5 per group. Data are presented as means ± SEM. Statistics by unpaired *t*-test.

To explore the impact of UGDH O-GlcNAcylation on NSCLC cells, CRISPR/Cas9-mediated deletion of UGDH was performed in A549 cells (KO cells) (Fig. 2A). Meanwhile, recombinant WT or S350A UGDH was reintroduced back to the KO cells (Fig. 2B). No apparent difference was observed in cell proliferation or cell viability between WT and UGDH-KO cells (Fig. S1F–J). In contrast, the cell migration was significantly inhibited in UGDH-KO cells (Fig. 2C, D). Furthermore, mice injected with UGDH-KO cells exhibited fewer lung metastatic nodules (Fig. 2E, F) and higher overall survival rates (Fig. 2G) compared to the control mice. This phenotype was also completely restored upon rescue with the WT, but not S350A UGDH (Fig. 2C–G).

To further investigate the functional impact of UGDH O-GlcNAcylation on tumor in vivo, mouse LLC cell lines stably expressing WT or S350A UGDH (Fig. S1K) were subcutaneously inoculated in parallel into syngeneic (C57BL6/J) and immunodeficient mice (C57BL6/J^nu/nu^), and the tumor growth and survival were monitored (Fig. 2H). In immunodeficient mice, LLC cells expressing WT or S350A UGDH exhibited similar tumor growth curves (Fig. 2I, left panel) and overall survival rates (Fig. 2J, upper panel). In contrast, syngeneic immunocompetent mice inoculated with LLC cells expressing S350A UGDH showed a lower tumor burden (Fig. 2I, right panel, and 2K) and prolonged overall survival (Fig. 2J, lower panel) compared with those inoculated with cells expressing WT UGDH. Moreover, to determine whether these observations were specific to lung cancer, we analyzed pancreatic cancer growth by inoculating Panc02 cells expressing WT or S350A UGDH in mice. Similar phenotypes were observed in the primary tumor growth and overall survival between immunodeficient and immunocompetent mice (Fig. S2), suggesting that the UGDH O-GlcNAcylation effect is general in different tumors. The notable difference in tumor growth observed in WT mice compared to nude mice was probably due to the different ages of mice used in the study. Collectively, these results revealed that O-GlcNAcylation promotes UGDH-mediated tumor growth and metastasis in vitro and vivo, and that the immune system is involved in regulating the tumor development.

### UGDH O-GlcNAcylation impacts the tumor immune cell landscape

**Fig. 3:**
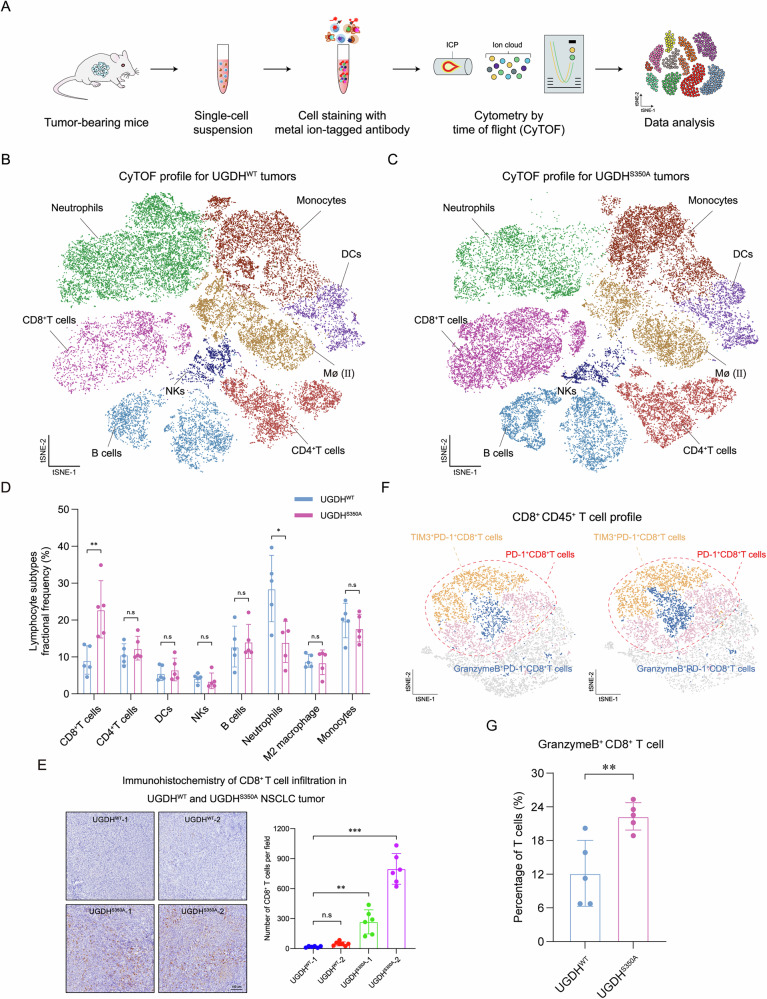
UGDH O-GlcNAcylation impacts the tumor immune cell landscape. **A** A flow chart illustrates tissue processing and CyTOF analysis of lung tumors generated by subcutaneous injection. Two-dimensional T-distributed stochastic neighbor embedding (t-SNE) scatterplot of the CyTOF-TILs data derived from UGDH-WT (**B**) and UGDH-S350A (**C**) tumor showed that 8 main clusters were identified with corresponding markers. **D** Comparing the contributions of various clusters of immune cells from total CD45^+^ lymphocytes between the two groups of samples. **E** Representative immunohistochemistry images and quantification of CD8^+^ T cells in primary tumors formed by UGDH-WT and UGDH-S350A NSCLC cells. Scale bar, 100 μm. **F** t-SNE re-analysis of CD8^+^ T cells colored by relative expression of CyTOF markers, with clusters indicated. **G** Comparing the percentage of Granzyme B^+^CD8^+^ T cells in lymphocytes between UGDH-WT and UGDH-S350A tumors.

To clarify the potential immune mechanism of UGDH O-GlcNAcylation on tumor development, we isolated immune cells from LLC tumors expressing WT or S350A UGDH, and subjected cells to cytometry by time-of-flight (CyTOF) [20] for a comprehensive profiling of the tumor immune microenvironment (TIME) (Fig. 3A). The CyTOF panel consisted of 37 cell membrane, intracellular, and nuclear markers, encompassing both established immune cell-surface markers as well as relevant effectors and immune checkpoint molecules (Table S1) [21]. All CyTOF data underwent pre-processing, and the analysis was focused solely on live single immune cells (CD45^+^), which were retained after gating. Two-dimensional (2D) t-SNE plots were generated to illustrate the distribution of immune lineages, facilitating a visual comparison. By assessing the expression levels of cell markers, we identified 8 main cell clusters, including CD4^+^ T cells, CD8^+^ T cells, NK cells, neutrophils, B cells, and other subpopulations such as dendritic cells, M2 macrophages, and monocytes (Fig. 3B, C). Cell clustering was conspicuous, indicating no abnormal effects during the experiments (Fig. S3). After analyzing the proportion of different cell clusters, we observed that tumors expressing S350A UGDH exhibited a significant enhancement of CD8^+^ T cell abundance compared to tumors expressing WT UGDH (Fig. 3D). Consistently, the immunohistochemistry (IHC) analysis also showed higher infiltration of CD8^+^ T cells in tumors expressing S350A UGDH, compared with tumors expressing WT UGDH (Fig. 3E). We also observed an apparent decrease in neutrophil population in tumors expressing S350A UGDH (Fig. 3D). The possible reason will be discussed in the “Discussion” section.

To further investigate the composition of CD8^+^ T cells in tumors, we extracted and reanalyzed CD8^+^ T cells and ultimately divided T cells into 8 clusters based on the molecular markers (Fig. S4). By detecting the abundance of classical effector including Granzyme B and checkpoint molecules such as PD-1, TIM, and LAG-3, we observed that cluster 14, classified as effector CD8^+^ T cells, was more enriched in tumors expressing S350A UGDH (Fig. 3F). Meanwhile, we found more Granzyme B^+^ CD8^+^ T cells in tumors expressing S350A UGDH compared with those expressing WT UGDH (Fig. 3G). Taken together, the results suggest that impairment of O-GlcNAcylation on UGDH remodels the TIME by regulating the infiltration of cytotoxic CD8^+^ T cells.

### UGDH O-GlcNAcylation promotes HA biosynthesis and remodels the ECM

**Fig. 4:**
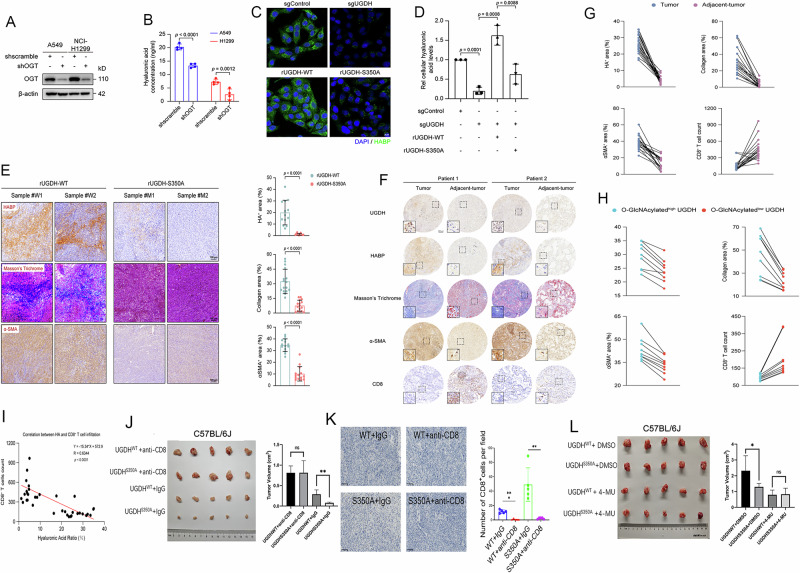
UGDH O-GlcNAcylation promotes HA biosynthesis and remodels the ECM. **A** Immunoblotting analysis of endogenous OGT depletion using shRNAs in A549 and NCI-H1299 cells. **B** ELISA analysis of HA concentrations in genetically engineered cells. **C** Immunofluorescence staining of HA accumulation from A549 cells upon UGDH depletion. HABP (HA stain in green) and DAPI (nuclear stain in blue). Scale bar, 10 μm. **D** Competitive ELISA analysis of the absolute concentration of HA in A549 cells with or without UGDH depletion. **E** Pathological staining and quantitative statistical analysis of the ECM contents. Masson’s trichrome staining for collagen or IHC staining for HA and α-SMA. The statistical data are shown as mean ± SEM (*n* = 12–16). Statistical analyses were performed by an unpaired two-tailed Student’s *t*-test. **F** IHC analysis was performed with anti-UGDH, anti-HA, anti-collagen, anti-α-SMA, and anti-CD8 antibodies on specimens from patients with lung cancer. Representative images of IHC staining graphs from two patients are shown. Scale bar, 200 μm. **G** A higher level of UGDH O-GlcNAcylation, α-SMA, collagen, HA, and a lesser infiltration of CD8^+^ T cells in tumor tissues were observed compared to adjacent peritumoral tissues in a cohort of 18 patient samples. **H** The deposition of HA, α-SMA, and collagen increased with the level of UGDH O-GlcNAcylation, but not for CD8^+^ T cells. **I** The correlation between CD8^+^ T cell infiltration and the expression of HA. **J** The effect of CD8α mAb treatment on tumor growth. **K** Representative IHC images and quantification of CD8^+^ T cells in the tumors after CD8α mAb treatment. Scale bar, 100 μm. **L** 4-MU treatment diminished HA accumulation and abolished the effect of UGDH O-GlcNAcylation on tumor development.

To further investigate the effect of UGDH O-GlcNAcylation on CD8^+^ T cell infiltration, we assessed the relationship between O-GlcNAcylation and the formation of HA. When OGT was depleted in A549 or H1299 cells, the cellular HA levels were significantly decreased (Fig. 4A, B). Moreover, UGDH KO significantly diminished the HA levels, which were completely restored by re-expression of WT, but not S350A UGDH, in cells (Fig. 4C, D). The tumors were further subjected to histopathological analysis to determine the ECM composition, which showed reduced collagen accumulation and expression of α-smooth muscle actin (αSMA) in tumors expressing S350A UGDH compared to those expressing WT UGDH, indicating a weaker desmoplastic phenotype (Fig. 4E). After treatment with exogenous HA, the migration ability increased in both UGDH KO and S350A variants compared to the control in A549, LLC, and Pan02 cells (Fig. S5A–C). In contrast, treatment with exogenous HA at the same concentrations did not have a significant impact on the colonization of these cells (Fig. S5D–F). The pulse-field gel electrophoresis showed reduced levels but similar sizes of HA produced from A549 cells expressing WT UGDH compared to cells expressing S350A UGDH (Fig. S5G).

The relationship of UGDH O-GlcNAcylation, αSMA, HA, and CD8 was further analyzed in 18 pairs of NSCLC samples with Masson’s trichrome staining (Fig. 4F, G). Compared to adjacent peritumoral tissues, tumoral tissues showed a higher level of UGDH O-GlcNAcylation, α-SMA, collagen, and HA, as well as a lesser infiltration of CD8^+^ T cells (Fig. 4G). In addition, the deposition of HA, α-SMA, and collagen increased with the level of UGDH O-GlcNAcylation, but CD8^+^ T cells did the opposite (Fig. 4H). Moreover, a strong correlation between CD8^+^ T cells infiltration and the expression of HA was observed (Fig. 4I). These results support the notion that UGDH O-GlcNAcylation regulates HA production to affect CD8^+^ T cells infiltration.

To further verify whether the UGDH O-GlcNAcylation-mediated antitumor effect is mainly dependent on CD8^+^ T cells, we employed a CD8α monoclonal neutralizing antibody (mAb) to deplete CD8^+^ T cells and an immunoglobulin G (IgG) isotype CTRL (IgG2b) as a control in C57BL/6 mice. In both the UGDH WT and UGDH S350A groups, CD8α mAb treatment promoted tumor growth moderately compared to the IgG2b treatment. However, CD8α mAb treatment largely diminished the population of CD8^+^ T cells in mice and significantly attenuated the influence of O-GlcNAcylation on tumor growth (Fig. 4J, K). Furthermore, 4-MU treatment significantly slowed tumor growth, with smaller tumor sizes and masses, which almost abolished the effect of UGDH O-GlcNAcylation on tumor development (Fig. 4L). Taken together, these results suggest that UGDH O-GlcNAcylation promoted tumor growth in a manner dependent on CD8^+^ T cell suppression through HA.

### O-GlcNAcylation enhances the enzymatic activity of UGDH

**Fig. 5:**
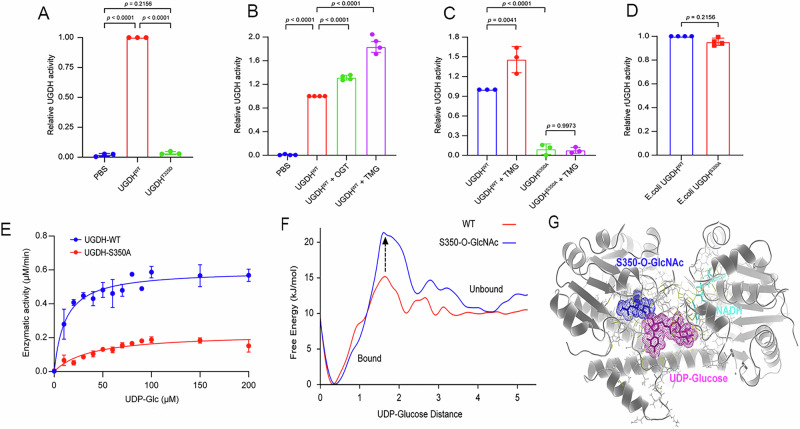
O-GlcNAcylation enhances the enzymatic activity of UGDH. **A** In vitro establishment of UGDH enzymatic reaction (*n* = 3). PBS group as a blank control, and the enzyme dead mutant UGDH^T325D^ as a negative control. **B** Enzymatic activity of UGDH in the presence of OGT co-expression or TMG treatment (50 μM) in HEK293T cells (*n *= 4). **C** Comparison of the enzyme activity of UGDH^WT^ and UGDH^S350A^ isolated from HEK293T cells in the presence of 50 μM TMG (*n* = 3). **D** Comparison of the enzyme activity of UGDH^WT^ and UGDH^S350A^ isolated from *Escherichia coli* (*n* = 4). **E** Steady-state kinetics of UGDH^WT^ and UGDH^S350A^ isolated from HEK293T for UDP-glucose (*n* = 3). **F** The free energy profile as a function of the distance of UDP-glucose (UDP-Glc) from the binding sites, as obtained from the ten one-microsecond simulations. **G** The representative structure model of O-GlcNAcylated UGDH. The S350-O-GlcNAc and UDP-glucose are depicted in sticks, while their molecular surfaces are illustrated by dots in blue and magenta, respectively. NADH is represented in green sticks, and UGDH is shown in white cartoon form. Data are presented as means ± SEM. Statistical analyses were performed by an unpaired two-tailed Student’s *t*-test.

We next explored the functional effect of O-GlcNAcylation on UGDH. The Ser350 is located within the UDP-binding domain of UGDH, suggesting a potential impact of Ser350 glycosylation on UGDH enzymatic activity. To test this hypothesis, we separately expressed Flag-tagged WT UGDH and a previously reported enzymatically dead mutant (T325D) in HEK293T cells [22]. The tagged proteins were purified with Flag-M2 beads for the subsequent activity analysis (Fig. 5A). We then investigated the effect of Ser350 O-GlcNAcylation on UGDH activity. Co-expression of OGT or the treatment with TMG markedly enhanced the enzymatic activity of UGDH compared to the control, indicating that O-GlcNAcylation promotes UGDH activity (Fig. 5B). Consistently, the S350A mutant exhibited a significant reduction in enzymatic activity compared to the WT. Treatment with TMG did not have an apparent effect on the activity of S350A UGDH (Fig. 5C). To exclude the possibility that the S350A mutation reduced UGDH activity due to the structural perturbation, we independently expressed and purified UGDH WT and S350A proteins in *Escherichia coli*. No apparent difference in the enzymatic activity was observed between bacterially expressed WT and S350A UGDH (Fig. 5D), suggesting that the reduced activity of the S350A mutant from HEK293T cells is due to the lack of glycosylation. To quantitatively analyze the enzymatic activity, we performed steady-state kinetics analysis of UGDH-catalyzed reactions (Fig. 5E). The Michaelis-Menten equation for the substrate UDP-glucose yielded a *K*_m_ value of 37.7 μM for the S350A, which was 3-fold higher than that of the WT with a *K*_m_ value of 11.4 μM (Fig. S6A). The maximum reaction rate (*V*_max_) for the WT (0.597 μM·min^−^^1^) was 2-fold higher than the S350A (0.224 μM·min^−^^1^). Conversely, the catalytic efficiency (*K*_cat_/*K*_m_) of the WT (5.07 s^−^^1^·μM^−^^1^) was 8-fold higher than that of the S350A (0.576 s^−^^1^·μM^−^^1^), suggesting that O-GlcNAcylation could increase the affinity of UGDH for the substrate UDP-glucose, thereby enhancing the enzymatic activity.

To gain a better understanding of the structural basis that underlies glycosylation-mediated UGDH activity, we carried out extensive all-atom molecular dynamics simulations of UGDH bound to its substrate in both non-glycosylated (WT) and glycosylated forms [23, 24]. For each form, we performed ten one-microsecond simulations, totaling 20 µs of simulation time. Our simulations unveiled that in the glycosylated form, UDP-glucose demonstrates heightened stability within the binding site compared to the WT (Fig. S6B). This enhanced stability correlates with an increased number of contacts formed between Ser350 and UDP-glucose (Fig. S6C). Particularly noteworthy is the observed significant rise in the unbinding free energy barrier (up to 7 kJ/mol) for the glycosylated form, as depicted in the free energy profile (Fig. 5F). This underscores the crucial role of S350-O-GlcNAcylation in reinforcing the stability of UDP-glucose binding. In the glycosylated form, the interaction between UGDH and UDP-glucose displays enhanced resilience and a prolonged duration compared to the non-glycosylated state. This heightened stability of UDP-glucose binding in the glycosylated form aligns with the observed increase in enzymatic activity as shown in enzymatic assays. Our simulations offer atomic-level insights into the specific molecular interactions driving these effects. The O-GlcNAcylation at Ser350 introduces favorable hydrogen-bonding interactions, thereby strengthening the binding interface between UGDH and UDP-glucose (Fig. 5G). These results contribute valuable insights into the molecular mechanism through which O-GlcNAcylation positively regulates UGDH enzymatic activity.

### UGDH O-GlcNAcylation reduces CD8 T cell infiltration by suppressing IFNγ-induced chemokine CXCL10

**Fig. 6:**
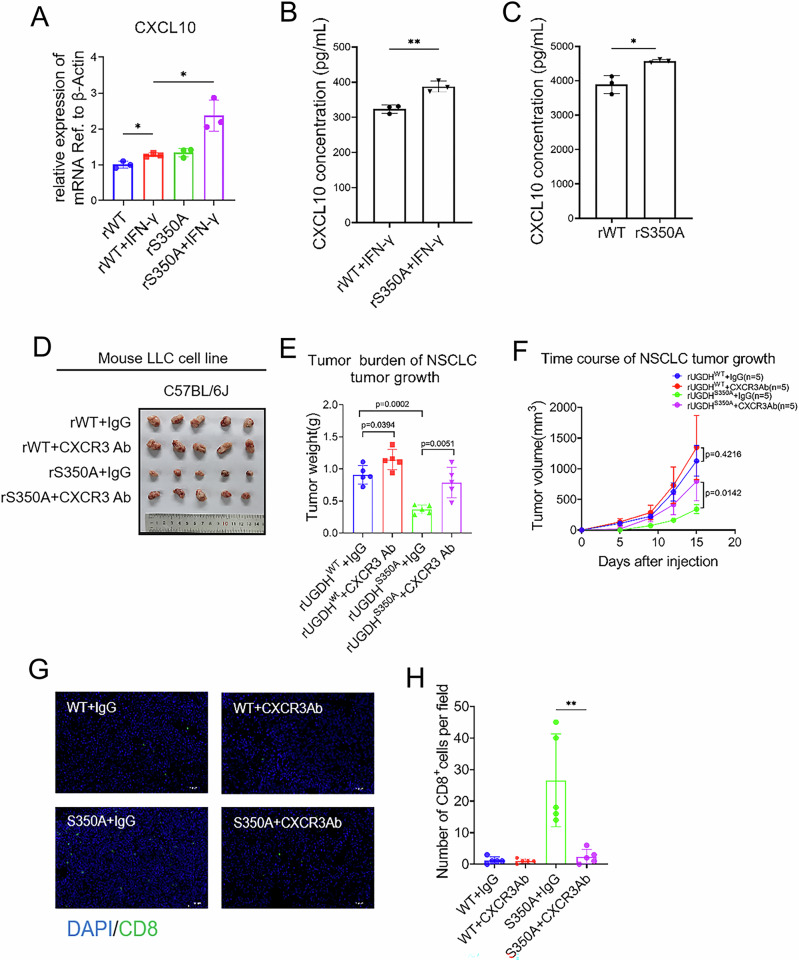
UGDH O-GlcNAcylation reduces CD8^+^ T cell infiltration by suppressing IFNγ-induced chemokine CXCL10. **A** The mRNA expression of CXCL10 in rWT and rS350A LLC cells treated with IFNγ (20 ng/mL) for 24 h (*n* = 3). **B** CXCL10 concentrations were measured by ELISA in rWT and rS350A LLC cells treated with IFNγ (20 ng/mL) for 24 h (*n* = 3). (**C**) CXCL10 concentrations were measured by ELISA in rWT and rS350A LLC tumors (*n* = 3). **D**–**H** The rWT and rS350A LLC cells were subcutaneously injected into C57BL/6 mice. Tumor-bearing mice were treated with IgG or CXCR3 Ab (10 mg/kg) every 4 days after inoculation. Images of the dissected tumors (**D**), tumor weight measured at the end of the experiment (**E**), tumor growth measured at the indicated time points (**F**), immunofluorescence staining (**G**) and quantification (**H**) of CD8^+^ T cell infiltration. Data are presented as means ± SEM. Statistical analyses were performed by an unpaired two-tailed Student’s *t*-test.

The infiltration of CD8^+^ T cells normally requires chemokines, and it has been reported that chemokines CCL2, CCL5, CCL8, CXCL9, and CXCL10 could recruit CD8^+^ T cells to the TIME [25–27]. We therefore quantified the mRNA expressions of CCL2, CCL5, CCL8, CXCL9, and CXCL10 in UGDH WT and UGDH S350A cells with or without IFNγ treatment. The results showed that CXCL10 levels were much higher in IFNγ-treated UGDH S350A cells than in UGDH WT cells (Figs. 6A, S7A). The O-GlcNAcylation of UGDH inhibited the expression of CCL8 and CXCL9, but showed no effect on CCL2 or CCL5. Furthermore, we observed a higher concentration of CXCL10 in the cell culture expressing S350A UGDH compared to that expressing WT UGDH after IFNγ treatment by ELISA (Fig. 6B). We also measured the concentration of CXCL10 in tumor tissues, and observed a similar upregulated CXCL10 expression in tumors expressing S350A UGDH (Fig. 6C).

The binding of CXCL10 to its ligand CXCR3 promotes the recruitment of T cells to the TIME. We used a CXCR3-specific antibody to block the CXCL10-CXCR3 interaction in UGDH S350A tumors to further confirm the involvement of CXCL10 in restraining UGDH S350A tumors. As expected, the treatment of the CXCR3-specific antibody largely restored the growth of UGDH S350A tumors (Fig. 6D–F) and decreased the infiltration of CD8^+^ T cells (Fig. 6G, H). These results suggest that O-GlcNAcylation of UGDH not only promotes HA production to remodel the ECM but also suppresses chemokine CXCL10 secretion to decrease CD8^+^ T cell infiltration.

### UGDH O-GlcNAcylation suppresses the nuclear translocation of STAT1

**Fig. 7:**
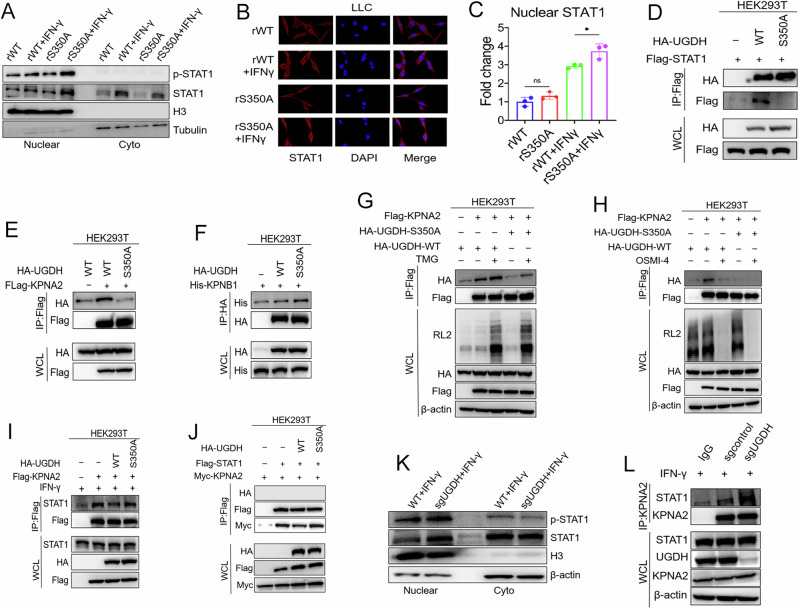
UGDH O-GlcNAcylation suppresses the nuclear translocation of STAT1. **A** Nuclear and cytoplasmic expression of STAT1 and p-STAT1 in UGDH WT and UGDH S350A LLC cells with or without IFNγ treatment. **B**, **C** STAT1 nuclear localization and quantification in rWT and rS350A LLC cells with or without IFNγ treatment (*n* = 3). **D** Immunoblotting analysis of the association of HA-tagged UGDH with Flag-tagged STAT1. Immunoblotting analysis of the association of Flag-tagged KPNA2 (**E**) and His-tagged KPNB1 (**F**) with HA-tagged UGDH in HEK293T cells. **G**, **H** Immunoblotting analysis of the association of KPNA2 with UGDH WT and UGDH S350A in HEK293T cells in the presence of TMG or OSMI-4. **I** Immunoblotting analysis of the association of Flag-tagged KPNA2 with the endogenous STAT1 in HEK293T cells in the presence of UGDH WT or UGDH S350A. **J** Immunoblotting analysis of the association of Myc-tagged KPNA2 and Flag-tagged STAT1 in HEK293T cells in the presence of HA-UGDH WT or HA-UGDH S350A. **K** Nuclear and cytoplasmic STAT1 and p-STAT1 expressions in UGDH WT or UGDH-KO cells after IFNγ treatment. **L** Immunoblotting analysis of the association of endogenous STAT1 and KPNA2 in UGDH WT and KO cells with IFNγ treatment. Data are presented as means ± SEM. Statistical analyses were performed by an unpaired two-tailed Student’s *t*-test.

We next explored the mechanism by which UGDH O-GlcNAcylation downregulates CXCL10 expression. It has been reported that STAT1 can transcriptionally upregulate CXCL10 expression. Notably, we observed higher levels of the phosphorylated form of STAT1 (transcriptionally active) in the nuclear extract in UGDH S350A cells compared with UGDH WT cells when treated with IFNγ (Fig. 7A). The immunofluorescence staining revealed consistent results (Fig. 7B, C). However, co-immunoprecipitation (Co-IP) assays revealed that UGDH only weakly interacted with STAT1 (Fig. 7D), suggesting that UGDH may not directly regulate STAT1. To further unravel the link between UGDH and STAT1, we performed immunoprecipitation coupled with mass spectrometry (IP-MS) to identify differential UGDH-interacting proteins between WT and S350A UGDH. Among the WT UGDH-interacting only proteins, we found KPNA2 and KPNB1, which are importins previously demonstrated to interact with STAT1 to mediate its nuclear translocation [28, 29]. Co-IP assays showed that KPNA2 interacted strongly with WT UGDH than with S350A UGDH, while KPNB1 interacted with WT and S350A UGDH at comparable levels (Fig. 7E, F), suggesting that O-GlcNAcylation regulates UGDH interaction with KPNA2 but not KPNB1. We therefore focused on KPNA2 in the following study. Treatment with TMG enhanced the association of KPNA2 with WT but not S350A UGDH (Fig. 7G). Consistently, treatment with OSMI-4 reduced the association of KPNA2 with WT but not S350A UGDH (Fig. 7H). To further analyze the effect of O-GlcNAcylation of UGDH on STAT1 and KPNA2 interaction, we ectopically expressed HA-tagged WT or S350A UGDH and Flag-tagged KPNA2 in HEK293T upon IFNγ treatment. A reduced amount of STAT1 was pulled down in cells expressing UGDH WT compared with cells expressing UGDH S350A (Fig. 7I). IP experiments also showed consistent results when performed in the reverse order (Fig. 7J). We speculate that UGDH likely competes with STAT1 to interact with KPNA2. Interaction of UGDH with KPNA2 reduces the amount of KPNA2 available for interacting with STAT1. In support of this hypothesis, more endogenous STAT1 was pulled down in UGDH-KO cells than in UGDH WT cells (Fig. 7K), and consistently more STAT1 and p-STAT1 were detected in the nuclear extract in UGDH-KO cells (Fig. 7L).

**Fig. 8:**
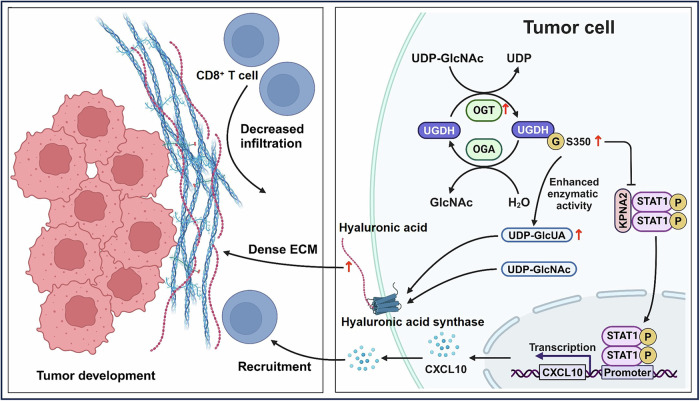
Schematic model of how O-GlcNAcylation of UGDH contributes to immune regulation and tumor development.

To further verify whether STAT1 directly regulates CXCL10 expression, STAT1 expression was depleted in LLC cells and forced-expressed in A549 cells, respectively. Accordingly, CXCL10 mRNA expression was significantly decreased upon STAT1 downregulation, and increased upon STAT1 upregulation (Fig. S7B–E). Dual-luciferase reporter assay showed the effects of STAT1 expression on promoting relative CXCL10-promoter activity in HEK293T cells (Fig. S7F). In addition, the chromatin immunoprecipitation (ChIP) assay demonstrated an enrichment of STAT1 at the CXCL10 promoter, compared to the IgG control (Fig. S7G). These results demonstrate that STAT1 transcriptionally regulates CXCL10 mRNA expression in cells. Taken together, these data indicate that O-GlcNAcylation increases UGDH-KPNA2 interaction to reduce KPNA2’s binding to STAT1, impairing its ability to mediate STAT1 nuclear translocation, thus leading to suppressed CXCL10 expression (Fig. 8).

## Discussion

Immunotherapy elicits tumor cell destruction by bolstering the body’s antitumor immune response. Immune checkpoint blockade (ICD) restores immune response by targeting negative regulatory molecules such as CTLA-4, PD-1, or LAG-3, thereby effectively suppressing tumor growth. However, only approximately 30% of cancer patients benefit from it, while the majority of patients remain unresponsive to the treatment [30, 31]. Over the past few decades, it has been established that chronic inflammation in the TME significantly contributes to tumor immune suppression [32]. The tumor-associated ECM, as the primary component of TME, promotes the growth, invasion, metastasis, and angiogenesis of tumors, while impeding cell death and drug diffusion [33]. Additionally, it plays a crucial role in tumor immune regulation, including interfering with tumor antigen presentation [34], affecting the initiation and activation of effector T cells [35–37], regulating T cell migration [38, 39], and impeding T cell recognition and destruction of cancer cells [40]. Hence, targeting the TME seems feasible for enhancing tumor immunotherapy.

The study identified the O-GlcNAcylation of UGDH as an important regulator of tumor invasiveness and tumor immunity. Decreasing UGDH O-GlcNAcylation weakens the migratory capability of tumor cells in vitro and in vivo. UGDH O-GlcNAcylation increased the intracellular content of HA and remodeled the ECM. UGDH is the pivotal rate-limiting enzyme in the uronic acid metabolism, which catalyzes the conversion of UDP-glucose to UDP-glucuronic acid, a precursor for the synthesis of HA. Our data demonstrated that O-GlcNAcylation on S350 within the UDP-binding domain markedly enhanced UGDH’s affinity for UDP-glucose, leading to increased enzymatic activity. Molecular dynamics simulations further illustrate that O-GlcNAcylation enhances the stability of UGDH binding to UDP-glucose. Besides, O-GlcNAcylation was reported to play a critical role in modulating protein stability, subcellular localization, and interactions. HuR, an mRNA stabilizing protein correlating with tumor metastasis, was previously reported to interact with UGDH [41]. Moreover, phosphorylated UGDH consumes UDP-Glc, diminishing its inhibitory effect on the binding of HuR to SNAI1 mRNA, consequently augmenting the stability of SNAI1 mRNA and protein expression, ultimately fostering tumor metastasis. However, Co-IP assays showed that O-GlcNAcylation had no appreciable impact on the interaction between HuR and UGDH (Fig. S8A). We also conducted an immunofluorescence assay to detect the subcellular localization of UGDH upon modulation of O-GlcNAcylation levels in cells. The distribution of UGDH in the nucleus and cytosol was not affected under different levels of O-GlcNAcylation (Fig. S8B, C) [42]. In addition, O-GlcNAcylation had a minimal effect on the half-life of UGDH, indicating that O-GlcNAcylation does not contribute to UGDH protein stability (Fig. S8D, E). These findings introduced a post-translational O-GlcNAcylation-dependent mechanism for regulating UGDH enzymatic activity within the TME.

In addition, O-GlcNAcylation of UGDH restricts the recruitment of CD8^+^ T cells, enabling solid tumors to escape immune surveillance. Experimental tumor models using genetically modified cell lines based on the UGDH gene revealed this ability, all of which expressed endogenous UGDH at high levels. O-GlcNAcylation of UGDH provides an obstacle to the recruitment of immune cells. Therefore, we conducted an in-depth analysis of differences in immunophenotypes using CyTOF and cell annotation. Compared with UGDH WT tumors, the loose ECM and more activated CD8^+^ T cells were present in UGDH S350A tumors with O-GlcNAcylation deficiency. We also observed an apparent decrease in neutrophil population in UGDH S350A tumors. The decrease in the neutrophil population might be attributed to the reduced HA production. As previously reported, CD44 was the main receptor of HA, and CD44/HA interaction was required for neutrophil adhesion in the liver microenvironment [43]. The reduced production of HA in tumor cells expressing S350A UGDH likely diminished CD44/HA interaction, leading to reduced infiltration of neutrophils in the TME.

Proinflammatory chemokines such as CCL2, CCL5, CCL8, CXCL9, and CXCL10 help tumor cells to recruit CD4^+^ and CD8^+^ T cells and improve T cell proliferation [44]. In our study, CXCL10 expression levels were significantly higher in IFNγ-treated UGDH S350A cells than in UGDH WT cells. It has been reported that STAT1 is a key regulator of CXCL10 transcription. Unexpectedly, IP-MS analysis showed that STAT1 only weakly interacted with UGDH. As a transcription factor, STAT1 needs importin for translocation into the nucleus. We therefore analyzed different importins interacting with WT or S350A UGDH in the IP-MS analysis, and demonstrated that O-GlcNAcylation increased UGDH’s binding to KPNA2 to compete against STAT1, thereby decreasing STAT1 translocation to the nucleus and the subsequent CXCL10 transcription.

Tumor cells are not the main source of chemokines. However, recent studies have shown that chemokines secreted by tumor cells also play a pivotal role in driving immunoregulatory functions [45]. For example, CXCL3 secreted by KRAS^G12D^ colorectal cancer cells promotes myeloid-derived suppressor cells migrating to the TME, which reduces the effectiveness of anti-PD-1 therapy [46]. CCL9 secreted by adenocarcinomas causes an influx of PD-L1^+^ macrophages and concurrent loss of B cells, T cells, and NK cells [47]. CXCL17 secreted by p53-deficient tumors mediates recruitment of immunosuppressive macrophages and Treg cells to the TME [48]. In line with these studies, our results demonstrate that CXCL10 produced by LLC cells expressing S350A UGDH can facilitate the recruitment of CD8^+^ T cells to suppress tumor growth. It is worth noting that other chemokines produced by other cell types in the TME likely contribute to the recruitment of CD8^+^ T cells.

As we have shown in our study that UGDH glycosylation plays a pivotal role in HA production and regulating tumor immunity in the TME, targeting UGDH glycosylation might provide an opportunity for enhancing the effectiveness of immunotherapy. One way to target UGDH glycosylation is to use an OGT inhibitor (such as OSMI-4) to reduce UGDH glycosylation. It has been reported that OGT inhibition combined with an anti-PD-L1 antibody can enhance immunotherapy effects [8, 9, 49]. On the other hand, to target UGDH glycosylation directly, it’s necessary to design peptides that contain the glycosylation site of UGDH. These peptides might competitively inhibit UGDH glycosylation without perturbing the global cellular glycosylation. It is also worth noting that we focused our study on NSCLC. As UGDH expression is significantly upregulated in multiple cancer tissues, including hepatocellular carcinoma and breast invasive carcinoma, it warrants investigating whether UGDH O-GlcNAcylation also plays a functional role in regulating tumor immunity in these types of cancer. In addition, UGDH is not the sole enzyme involved in HA biosynthesis. Hyaluronan synthase 2 (HAS2) is responsible for the polymerization and deposition of hyaluronan in the ECM. HAS2 is shown to be modified by OGT at Ser221, and O-GlcNAcylation of HAS2 prevents HAS2 degradation to enhance hyaluronan synthesis [50]. Whether HAS2 O-GlcNAcylation contributes to regulating tumor immunity via remodeling the TME remains to be elucidated.

In summary, our findings not only shed light on the regulatory mechanism governing UGDH biological function underlying tumor immunity and tumor growth, but also provide a potential strategy for improved cancer immunotherapy.

## Methods

### Materials

#### Antibodies

Antibodies used in this study were obtained from various companies. Specifically, the HRP-labeled monoclonal antibody against beta-actin (catalog number HRP-60008, 1:5000 dilution for immunoblotting) and the rabbit polyclonal antibody against UGDH (catalog number 13151-1-AP, 1:1000 dilution for immunoblotting) were purchased from Proteintech. The mouse monoclonal antibody against Flag (catalog number F1804, 1:5,000 dilution for immunoblotting), alpha-smooth muscle actin (catalog number A5228, 1:200 dilution for IHC), and biotinylated hyaluronic acid-binding protein (catalog number 385911, 1:1000 dilution for IHC or ELISA) were all acquired from Merck. The mouse monoclonal antibody RL-2 (MA1-072, 1:1000 dilution for immunoblotting), which recognizes O-linked N-acetylglucosamine (O-GlcNAc), was sourced from Invitrogen. The rabbit monoclonal antibodies against O-GlcNAc transferase (catalog number ab177941, 1:1,000 dilution for immunoblotting) and CD8-alpha (catalog number ab237723, 1:500 dilution for IHC) were purchased from Abcam. The rabbit monoclonal antibodies against hemagglutinin (HA) (catalog number 3724, 1:5000 dilution for immunoblotting) and CD8-α (catalog number 98941,1:500 dilution for IHC) were purchased from Cell Signaling Technology. The anti-CD8α antibody was obtained from Bio X Cell Inc. (catalog # BE0117, clone # YTS 169.4). The rabbit polyclonal antibody against UGDH (catalog number NBP1-90906, 1:200 dilution for immunofluorescence) was obtained from Novus. The clone number, quality label, and antigen location of each antibody used in the CyTOF experiment are shown in Supplementary Table S1.

#### Reagents

Flag-peptides and a protease inhibitor cocktail were both purchased from Merck. The cell transfection reagents polyethyleneimine (PEI) and polybrene were obtained from Yeasen. Antibiotics such as puromycin and blasticidin were obtained from Invivogen. Growth factors such as epidermal growth factor (EGF) and other small molecules, including Thiamet-G, β-NAD, β-NADH, and UDP-Glucose, were all purchased from MedChemExpress. Enzyme-linked immunosorbent assay (ELISA) kit for measuring intracellular hyaluronic acid was obtained from FineTest. The ClonExpress II One Step Cloning Kit was brought from Vazyme.

#### Plasmids

PCR-amplified human UGDH cDNA from Invitrogen’s cDNA library was inserted into multiple vectors, including p3×Flag-CMV7.1, pET28b-His, pLVX-HA-IRES, and pLVX-pmin-BSD. Additionally, a glycosylation site mutant of UGDH was created using site-directed mutagenesis kits.

The shRNA sequences of non-targeted (5′-GAACTCCGAGTTCACATGG-TTCCTCC-3′) and targeting human OGT coding region (5′-GCCTAAGTGAGT-CCAA-3′) were also utilized.

The single-guide RNA sequences (sgRNA) were employed to target the UGDH genome: 5′-CACCGGGGGGGGATGAAAACTCCAGA-3′ and 5′-CACCGAGAAGAGATATGATTGGAG-3′, both of which target exon 5 of the UGDH transcript.

### Cell culture and transfection

Human embryonic kidney 293T (HEK293T), A549, and NCI-H1299 cell lines were acquired from the American Type Culture Collection (ATCC) library. Lewis lung carcinoma (LLC) and Panc02 cells were kindly provided by the Liang laboratory. All cell lines were verified to be free of mycoplasma contamination. The abovementioned cell lines were maintained at a 37 °C incubator in 5% CO_2_ with Dulbecco’s Modified Eagle Medium (DMEM) supplemented with 10% fetal bovine serum (FBS).

For transfection, the indicated plasmid was mixed with polyethylenimine (PEI) in a 1:6 ratio in Opti-MEM medium and subsequently added to the target cells.

### O-GlcNAcylation labeling assay

The glycosylation labeling was conducted following previously reported methods. Briefly, O-linked GlcNAcylation within the cell lysate was labeled with azide via a reaction between UDP-GalNAz and β-Galactosyltransferase GalT^Y289L^. The resulting azide-labeled proteins were then conjugated to biotin via a copper-catalyzed azide-alkyne cycloaddition (CuAAC) reaction, commonly known as “click” chemistry. Biotinylated proteins were resuspended in a neutral buffer composed of 6% NP-40, 100 mM Na_2_HPO_4_, 150 mM NaCl, followed by overnight incubation at 4 °C with streptavidin resin. After thorough washing with low-salt buffer (100 mM Na_2_HPO_4_, 150 mM NaCl, 1% Triton-100, 0.5% sodium deoxycholate, 0.1% SDS, pH = 7.5), high-salt buffer (100 mM Na_2_HPO_4_, 500 mM NaCl, 0.2% Triton-100, pH = 7.5), and PBS, the bound proteins were boiled and eluted with protein loading buffer. Immunoblotting analysis was subsequently performed using an antibody against UGDH.

### Immunoprecipitation (IP) and site mapping of UGDH glycosylation

For IP or Co-IP protocols, cells were lysed with IP buffer (1% Tween-20, 150 mM NaCl, 50 mM Hepes, pH 7.8) or Co-IP buffer (0.4% NP-40, 150 mM NaCl, 50 mM Hepes, pH 7.8), and the lysates were incubated with anti-Flag/anti-HA magnetic agarose beads at 4 °C overnight. After IP, beads were washed three times with IP or Co-IP buffers and analyzed by Western blotting.

Recombinant plasmids encoding flag-tagged UGDH were transiently transfected into HEK293T cells and treated with Thiamet-G (50 μM) to enhance glycosylation levels. 48 h after transfection, the cells were harvested and homogenized, and the resulting supernatant was collected following centrifugation at 10,000 × *g* for 30 min. The supernatant was then mixed with anti-flag resin and allowed to incubate overnight at 4 °C. Purified UGDH was eluted with 0.5 mg/mL of Flag-peptide and subjected to 10% SDS-PAGE. The UGDH band was visualized using Coomassie Brilliant Blue R-250 staining and excised from the gel. The gel slices were destained with acetonitrile and rehydrated in 50 mM iodoacetamide (IAM) containing 10 mM dithiothreitol (DTT) at 56 °C for 45 min. The protein in the gel was digested with trypsin/GluC at 37 °C for 16 h. Following tryptic digestion, the peptides were desalted using C18 ZipTips (Millipore) and subjected to liquid chromatography-tandem mass spectrometry (LC-MS/MS) for glycosylation site mapping.

### In vitro enzymatic assay for UGDH

The enzymatic activity of UGDH was assessed following previously established protocols. Specifically, UGDH facilitates the conversion of two molecules of NAD^+^ and one molecule of UDP-Glc to produce two molecules of NADH and UDP-GlcUA. Thus, the activity of UGDH can be evaluated by measuring the change of OD_340_, which reflects the generation of NADH. This examination was carried out in 0.1 M sodium phosphate buffer (pH 7.4) at room temperature. To determine the Michaelis constant (*K*_m_) and maximum velocity (*V*_max_) of UDP-Glc, a fixed concentration of NAD^+^ was maintained, and the concentration of UDP-Glc was varied from 0 to 1 mM. Conversely, a constant concentration of UDP-Glc was maintained while the concentration of NAD+ was adjusted from 0 to 10 mM to determine the *K*_m_ and *V*_max_ of NAD^+^. Data from three replicates at each concentration were plotted using GraphPad Prism 9, and *K*_m_ and *V*_max_ were calculated via fitting the data to the Michaelis-Menten equation.

### CRISPR-Cas9-mediated knockout of UGDH

This study utilized lentivirus-mediated CRISPR-Cas9 technology to knock out *UGDH* in mammalian cells. The single-guide RNA (sgUGDH) sequence was retrieved from the public GeCKO v2 library. Annealed oligonucleotides were inserted into the pLentiCRISPRv2 vector, digested by *BsmB1*, which can co-express Cas9 protein and sgRNA simultaneously. Lentivirus particles were generated through co-transfection of HEK293T cells with the modified pLentiCRISPRv2 vector and packaging plasmids. Then, lentivirus-containing medium was collected 48- and 72-h post-transfection. Target cells were infected with the virus-containing medium mixed with complete medium at a ratio of 1:1 and supplemented with 20 μg/mL polybrene. Cells were selected with 2 μg/mL puromycin for one week to achieve complete cell elimination. Following screening, individual cells were seeded into 96-well plates, and upon reaching 80% confluence, were transferred to 24-well plates for expansion and assessment of UGDH protein levels via immunoblotting to establish stable UGDH knock-out cell lines.

### Generation of UGDH reconstituted stable cell lines

Human UGDH cDNA (WT and S350A) and mouse UGDH cDNA (WT and S350A) were inserted into pLVX-pmin-BSD vectors, and the corresponding lentivirus particles were generated. Then, endogenous UGDH-KO A549, LLC, and Panc02 cells were infected with the lentiviruses, and stable cell lines were selected with 10 μg/mL blasticidin for 2 weeks.

### Quantitative reverse transcription polymerase chain reaction (RT-qPCR)

Total RNA was extracted from cultured cells utilizing Trizol reagent (TaKaRa, 9109), and the quantity and integrity of RNA were confirmed using a nanodrop spectrophotometer. A total of 500 ng RNA was reverse transcribed into cDNA with the aid of PrimeScript™ RT reagent Kit (TaKaRa, RR047A) and diluted 10-fold for subsequent fluorescence-based qPCR analysis. The SYBR Green method was employed on a Bio-Rad CFX Connect instrument to measure cDNA levels. Relative expression levels of target genes were calculated using the 2^−ΔΔCt^ method with SDHA serving as an internal control.

### Dual-luciferase reporter assays

The vectors pCXCL10(human)-Fluc-SV40-hRluc (MIAOLING PLASMID, P75036) and pCMV-STAT1 were co-transfected into HEK293T cells at a ratio of 1:1. After 48 h of transfection, luciferase assays were performed using a Dual-Luciferase Reporter Gene Assay Kit (Yeasen, 11402ES60) according to the manufacturer’s instructions. The promoter activities were normalized to the corresponding values of Renilla luciferase.

### Chromatin immunoprecipitation assay (ChIP)

The vectors plvx-HA (control) or plvx-HA STAT1 were transfected into A549 cells for a duration of 48 h. The ChIP assay was performed using the Sonication ChIP Kit (Abclonal, RK20258) following the manufacturer’s protocol. A total of 8 μg anti-STAT1 antibody (Proteintech, 10144-2-AP) was used for IP. The primers for the CXCL10 gene promoter were as follows: (forward, 5′-TTTGGAAAGTGAAACCTAATTCA-3′; reverse, 5′-AAAACCTGCTGGCT-GTTCCTG-3′).

### Western blot analysis

Cells were rinsed with ice-cold PBS prior to being lysed in RIPA buffer supplemented with protease inhibitors. The concentration of total protein in the lysate was measured with a BCA protein quantification kit. Proteins were separated by SDS-PAGE and transferred to polyvinylidene fluoride (PVDF) membranes. Membranes were blocked in PBST containing 5% non-fat milk for 30 min before incubation with primary antibodies specific to the desired proteins overnight at 4 °C. Subsequently, membranes were washed three times with 0.05% PBST and incubated with horseradish-peroxidase (HRP) conjugated second antibody for 1 h at room temperature. Enhanced chemiluminescence (ECL) was employed to visualize HRP signals, and ImageJ software was used to analyze target band intensities for further analysis.

### Immunofluorescence

For immunofluorescence staining, an appropriate number of cells were plated onto coverslips in a 12-well plate and allowed to adhere overnight. Cells were washed in chilled PBS and fixed with 4% neutral formaldehyde for 30 min, followed by permeabilization with 0.2% Triton X-100 for 5 min. Cells were blocked with 3% BSA for 30 min at room temperature and subsequently incubated with primary antibodies diluted in 1% BSA at 4 °C overnight. Following washes with PBS, cells were incubated with fluorophore-conjugated secondary antibody in the dark for 1 h at room temperature. Coverslips were mounted onto slides with anti-fade mounting medium containing DAPI (Vector Laboratories) and imaged via confocal microscopy.

### Protein stability assay

To assess protein stability, Flag-tagged UGDH recombinant plasmids were transfected into HEK293T cells and cultured in medium containing 100 μg/mL cycloheximide (CHX). After 8 h of CHX treatment, cells were harvested at various time points (0, 2, 4, 8, 12, and 16 h) and subjected to sonication in RIPA buffer. The resulting supernatants were analyzed for protein concentration using a BCA protein assay kit, and protein samples were subjected to immunoblotting to evaluate changes in UGDH protein expression over time.

### Cell viability and colony formation assays

Cell viability assays were conducted using the Cell Counting Kit-8 (CCK-8) according to standard protocol. Briefly, 1000 cells were seeded into each well of a 96-well plate, and 10 μL CCK-8 solution was added to each well. Plates were incubated at 37 °C for 3 h before absorbance at 450 nm was measured. The proliferation rate was monitored daily for 5 days to generate a growth curve.

To assess anchorage-independent colony formation ability, cells were seeded into soft agar at a density of 5000 cells per well in 6-well plates and cultured for 2 weeks. Medium was refreshed every three days. Colonies were fixed with paraformaldehyde and stained with 0.5% crystal violet. Images were captured, and colonies were counted manually. Each experiment consisted of triplicate wells.

### Wound healing and transwell migration assays

3 horizontal lines were generated by a marker pen on the back of the 6-well plate, with a spacing of 0.5–1 cm between each horizontal line for easy positioning. Wounds were created in confluent monolayers of A549 cells grown in this 6-well plate using a pipette tip. Suspended cells were removed by washing with PBS before adding fresh medium. Images were captured at regular intervals to monitor wound closure. Quantitation of wound closure was performed using ImageJ software.

To examine migratory potential, 2 × 10^4^ A549 cells suspended in serum-free medium were seeded into the upper chamber of a Transwell system, while complete medium containing 10% FBS was added to the bottom chamber to create a nutrient gradient. Following 24 h of incubation at 37 °C, cells that had migrated to the underside of the membrane were fixed with 4% paraformaldehyde and stained with 0.4% crystal violet. Three independent repeated experiments were conducted on each type of cell, and images were taken in random five fields of view under a microscope.

### Bioinformatics analysis of the TCGA database

In this study, we employed the online platform GEPIA2 to conduct bioinformatic analysis of the TCGA database. Our initial step involved examining the mRNA expression levels of UGDH across 31 different types of cancer included in TCGA. This was followed by an investigation of the association between UGDH expression and tumor stage in lung adenocarcinoma patients. To further assess the clinical significance of UGDH, we utilized Kaplan–Meier survival analysis to evaluate its correlation with overall patient survival rates in lung adenocarcinoma.

### Hematoxylin & eosin (H&E) staining and immunohistochemistry

Tumor specimens were extracted from mice and fixed in 4% paraformaldehyde at room temperature for 24 h. The samples were then processed according to established laboratory protocols, including embedding in paraffin, sectioning into 6-micron-thick slices, and drying at 56 °C overnight. Tissue sections were dewaxed with xylene and dehydrated with gradient ethanol.

For hematoxylin & eosin (H&E) staining, these slices were submerged in hematoxylin for five minutes, followed by brief exposure to acid alcohol differentiation solution and bluing with ammonium hydroxide. Eosin solution was applied for three minutes, after which the slides underwent a dehydration process using a series of ethanol and xylene, ultimately leading to mounting with neutral resin and microscopic examination.

Immunohistochemical staining was performed by subjecting the sections to antigen retrieval in a citric acid buffer (pH 6.0) heated at 95 °C for 30 min or protease K treatment. Blocking was carried out with Dako serum blocker for 30 min at room temperature before incubating with primary antibodies overnight at 4 °C. Biotin-conjugated secondary antibodies were introduced for another 30-min incubation period, followed by the addition of 3,3′-diaminobenzidine (DAB) as a chromogenic substrate and the application of the avidin-biotin complex (ABC) method to visualize the target proteins. Hematoxylin counterstaining was performed following the final steps of the immunostaining procedure.

### CyTOF sample processing and data acquisition

The tumor tissue was excised into fragments and subsequently digested by Collagenase IV, DNase I, and Dispase II. After digestion, a 70 μm cell strainer was used to filter the tissue. Red blood cell lysis buffer and 30% Percoll were added, respectively, to remove red blood cells and cellular debris.

Single cell suspension was then strained with cisplatin to exclude dead cells and then incubated with Fc blocking solution at room temperature for 10 min. Cell-surface antibodies were then applied to stain the cells in cell staining buffer (Yeasen) for 30 min at room temperature. Intracellular antibodies were used to stain the cells after permeabilization. The total number of 37 metal-conjugated antibodies used for CyTOF in this study is listed in Supplementary Table 1. Finally, cells were stained with Intercalator-Ir overnight at 4 °C. The data were acquired using the Helio3 CyTOF Mass Cytometer (Fluidigm).

We used FlowJo software (v.10.8.1) to exclude debris, dead cells, and doublets by using step-by-step manual gating, leaving DNA+ cisplatin-CD45+ cells for subsequent analyses. R package Rtsne and ggplot2 were used to visualize the CyTOF data. The clustering of immune cells within and between samples is executed utilizing the R package Phenograph.

### ELISA for the detection of hyaluronic acid (HA)

Cellular extracts were prepared by lysing cells in chilled RIPA containing protease inhibitors. Place the cell lysate on ice for 30 min, sonicate and centrifuge (4°C, 10,000 × *g*, 30 min) to obtain the supernatant. The pre-coated ELISA plate was washed with wash buffer. Then Standard samples and cell extract samples were added to the pre-coated ELISA plate, followed by the addition of biotinylated hyaluronic acid-binding protein (HABP) and incubation at 37 °C for 45 min. Streptavidin-horseradish peroxidase (HRP) was introduced and allowed to react for another 30 min, after which tetramethylbenzidine (TMB) was used to induce colorimetric changes. Absorbance values were read at 450 nm, and the results were interpreted based on a standard curve to determine the HA content of each sample.

### ELISA for the detection of CXCL10

Concentrations of CXCL10 from cell culture supernatants or tissues were determined using an ELISA kit (Proteintech, KE00128) according to the manufacturer’s protocol. The concentrations of CXCL10 were calculated based on Optical Density (OD) values at a detection wavelength of 450 nm.

### Animal studies

The study involving animal experimentation obtained ethical approval (Reference Number: 2024-303) from the Committee of Animal Ethics at the First Affiliated Hospital of Zhejiang University. The mouse strains used in this study included C57BL/6J (Jackson Laboratory, Catalog No.000664) and C57BL/6J^nu/nu^ (B6.Cg-Foxn1nu/J, Jackson Laboratory, Catalog No.000819). In an aseptic facility, age-matched (8-week-old) male mice were bred and randomly and evenly grouped for subsequent experiments.

### Lung metastasis assay

In short, 1 × 10^5^ UGDH^WT^ or UGDH^S350A^ A549 cells suspended in 200 μL PBS were slowly injected into the tail vein of mice. Following the injection, the animals’ survival status is monitored every two days. Once the designated monitoring point has been reached or when the presence of tumor-related symptoms such as lethargy and labored breathing becomes apparent, the animals are euthanized, and their lung tissues are collected for further histological examination.

### Subcutaneous tumor model construction

For subcutaneous injection experiments, 2 × 10^6^ cells suspended in 100 μL PBS were injected into the same side of C57BL/6J and C57BL/6J^*nu/nu*^ mice. The growth of tumors in the injection site is monitored daily, and their diameters are measured using a caliper. Upon reaching the designated endpoint or when the tumor volume exceeds a predetermined limit, the animals are euthanized, and their tumor tissues are harvested for further pathological analysis.

### Molecular dynamics simulations of UGDH

The substrate-bound UGDH initial model was derived from the crystal structure (PDB, 6C5A). Explicit solvent all-atom simulations for UGDH in both non-glycosylated and glycosylated forms were generated using the CHARMM-GUI server. Specifically, in the glycosylated form, S350 was modified with an O-GlcNAc glycan. To neutralize the systems and mimic physiological ionic concentration (0.15 M), Na^+^ and Cl^−^ ions were added to the solvent. The CHARMM36m force field was applied for the complex and ions, and the TIP3P model represented waters. CGenFF force field parameters were used for UDP-glucose and NADH. Protonated states for residues were assigned based on ProPKA predictions, with Glu^128^ and Glu^165^ being protonated in our models. Long-range electrostatic interactions were managed using the particle mesh Ewald method. The cutoff distance for both Lennard-Jones and real-space Coulomb interactions was set at 12 Å. Covalent bonds involving hydrogen atoms of proteins and water molecules were constrained using the LINC algorithm. Each system underwent a 1000-step minimization, followed by equilibration in an NPT ensemble with position restraints on all heavy atoms of the complex. A time step of 2.0 fs was employed, and temperature was maintained at 300 K using the Nose–Hoover thermostat. Both non-glycosylated and glycosylated forms underwent ten repeats in molecular dynamics simulations using the GROMACS package (version 2022.5). Trajectories were analyzed using PLUMED.

### Mass cytometry analysis of tumor-infiltrating lymphocytes

Established tumors are surgically excised from mice and minced before being treated with RPMI-1640 medium containing 1 U/mL recombinant DNase I and 4 mg/mL Type IV Collagenase for 4 h at 37 °C. The resulting cell suspension is subjected to red blood cell lysis using ACK lysis buffer (150 mM NH_4_Cl, 10 mM KHCO_3_, 0.1 mM EDTA, pH 7.2–7.4), filtered through a 70 μm strainer, and separated using 30% Percoll density gradient centrifugation (4 °C, 450 g, 10 min) to remove cellular debris. Subsequently, ten million cells per sample are stained with cisplatin (0.5 μM), fixed, and cryopreserved in 10% DMSO/Serum at −80 °C.

Metal-conjugated antibodies are prepared following the manufacturer’s instructions (Fluidigm). Briefly, metal-labeled X8 polymers are conjugated to 100 mg of antibody. After metal conjugation, the concentration of each antibody was assessed by BCA quantification kit and adjusted to 0.5 mg/mL in Candor Antibody Stabilizer. Conjugated antibodies were titrated to obtain an optimal concentration for use.

The cells are blocked with FcR-blocking solution (BD) for 10 min at 4 °C and subsequently stained with a cell membrane antibody cocktail panel for 30 min at room temperature. After washing twice with CSB buffer, the cells are permeabilized using Fix I buffer (Fluidigm) for 20 min at room temperature. Intracellular staining is performed with an antibody cocktail panel for 30 min at room temperature, followed by another round of washing and incubation overnight with cell intercalation solution containing nucleic acid Ir-intercalator (Fluidigm). Finally, the cells are washed once with CSB and once with Cell Acquisition Solution (CAS) (Fluidigm) before being diluted to 1.1 × 10^6^ cells/mL in CAS containing 10% EQ Four Element Calibration Beads (Fluidigm) and analyzed on a Helios mass cytometer (Fluidigm) at a rate of less than 300 events per second. Data analysis is performed using Flowjo software to gate out debris, dead cells, and doublets, leaving only DNA^+^CD45^+^cisplatin^low^ cells for subsequent clustering and high-dimensional analyses. Arcsinh-transformed CyTOF data is visualized using t-distributed stochastic neighbor embedding (t-SNE), a dimensionality reduction technique that employs the Barnes-Hut algorithm and is implemented using the Rtsne package. The resulting t-SNE plots incorporate data from all samples and are used to cluster CD45-positive cells both within and between samples using the Phenograph package, which is based on the Louvain community detection algorithm. Heatmaps of the resulting clusters and markers are generated using the pheatmap package.

### Statistical analysis

Statistical analyses are conducted using Student’s *t*-test for comparing two sets of data and one-way ANOVA for comparing multiple groups (greater than two). Data is represented as mean ± standard error. Pearson correlation analyses and all other statistical calculations are performed using GraphPad Prism 9 and R 4.2.2, where *p* < 0.05 is deemed statistically significant.

## Supplementary information

Supplementary Figures

Uncropped Western blots

## Data Availability

All data and material used in this manuscript are available and can be requested from the corresponding authors.
