# Supplementary material for: O-GlcNAcylation of UGDH regulates its activity and remodels the extracellular matrix to facilitate tumor growth
Source: Cell Death Differ. 2025 Oct 6;33(3):574–88. doi: 10.1038/s41418-025-01591-8 (PMC13036090; doi:10.1038/s41418-025-01591-8)
Supplement: Supplementary file 1 — Supplementary Figures [file 41418_2025_1591_MOESM1_ESM.pdf]

Supporting Information

Supplementary Figures

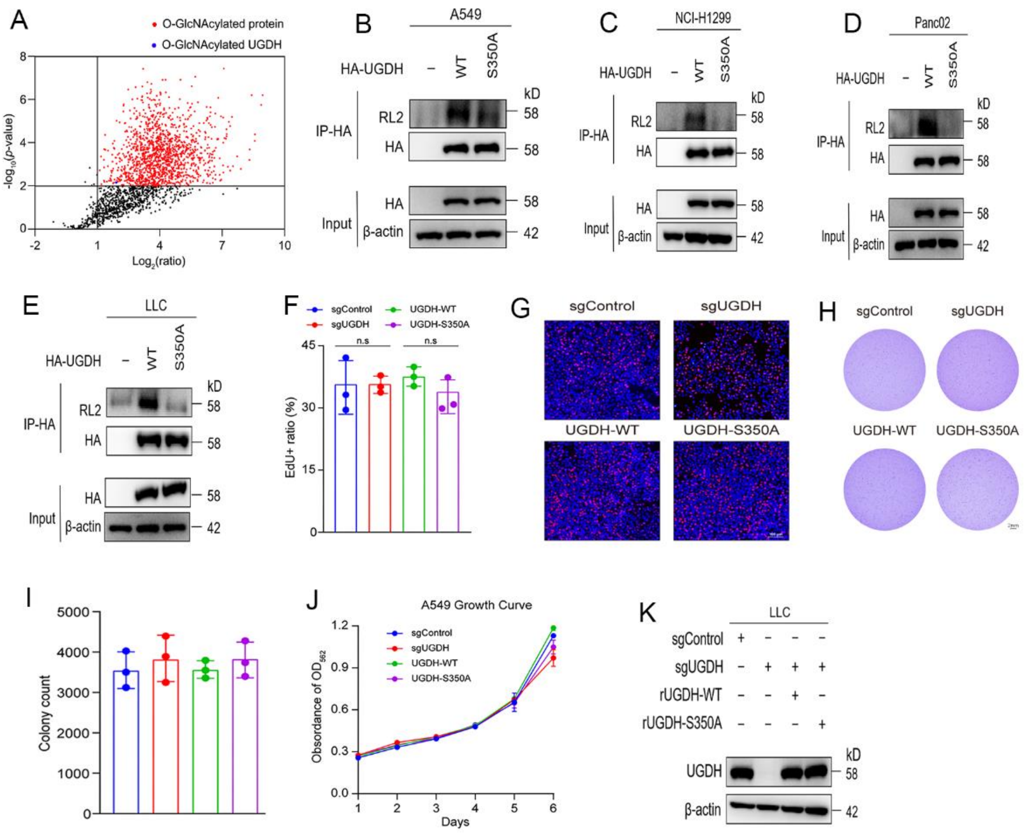

Fig. S1. O-GlcNAcylation of UGDH does not affect tumor cell proliferation.

(A). Mass Spectrometry profiling of O-GlcNAcylated proteins showing UGDH as a potential substrate with increased glycosylation levels.

(B-E). S350A mutation reduced the O-GlcNAcylation levels in A549 cells (B), NCI-H1299 cells (C), Panc02 cells (D), and LLC cells (E).

(F, G). The proliferation of engineered A549 cells detected with the EdU labeling experiment.

(H, I). Representative graphs (H) and quantification (I) of colonization ability of engineered modified A549 cells detected by soft agar assay.

(J). The growth curve of the engineered A549 cells by CCK-8 detection.

(**K**). Immunoblotting analysis of the basal level, ectopically expressed WT and S350A UGDH in LLC cells.

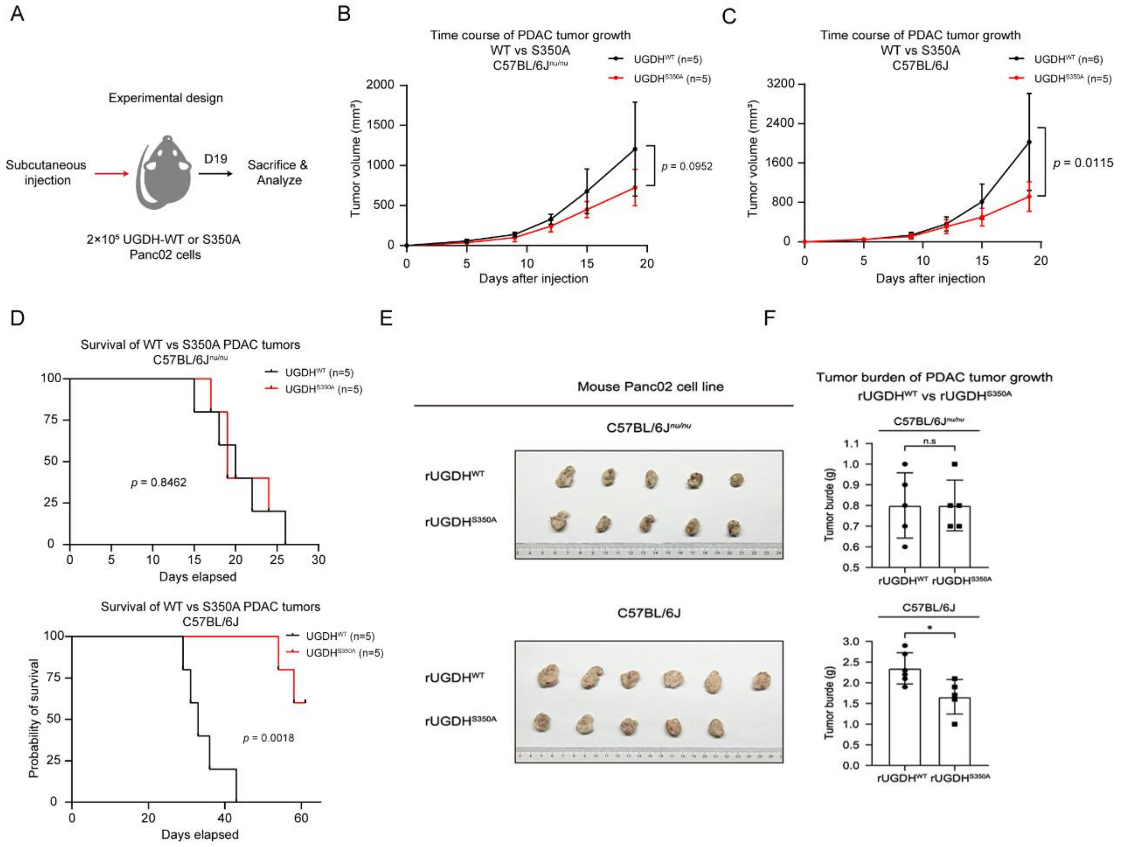

Fig. S2. UGDH O-GlcNAcylation promotes pancreatic cancer development and improves survival in immunocompetent mice.

(A). Schematic diagram of subcutaneous injection of pancreatic cancer cells and detection of primary tumor growth. In brief, Panc02$^{UGDH\text{-}WT}$ and Panc02$^{UGDH\text{-}S350A}$ cancer cells were subcutaneously injected into the right side of C57BL/6J immunocompetent and C57BL/6J $^{nu/nu}$ immunodeficient mice. On day 19, all mice were sacrificed and tumors were collected for further analysis.

(B-C). Tumor growth curves of engineered UGDH-WT and UGDH-350A PDAC tumors in C57Bl/6J$^{nu/nu}$ (B) or C57BL/6J (C) mice (n = 5 mice per group). Statistics by student's t-test. Tumor volumes were measured three times per week and calculated using the formula $V = \pi/4 \times \text{length} \times \text{width}^2$.

(D). Overall survival (OS) of C57Bl/6J$^{nu/nu}$ or C57BL/6J mice bearing primary tumor seeded with UGDH-WT or UGDH-350A cells (n = 5 mice per group). Statistics by Kaplan-Meier test.

(E-F). Tumor graphs (E) and weights (F) of PDAC UGDH-WT and UGDH-S350A

growing in C57BL/6J$^{nu/nu}$ or C57BL/6J mice at day 19 after s.c. injection (n = 5 per group). Data are presented as means ± SEM. Statistics by unpaired t-test.

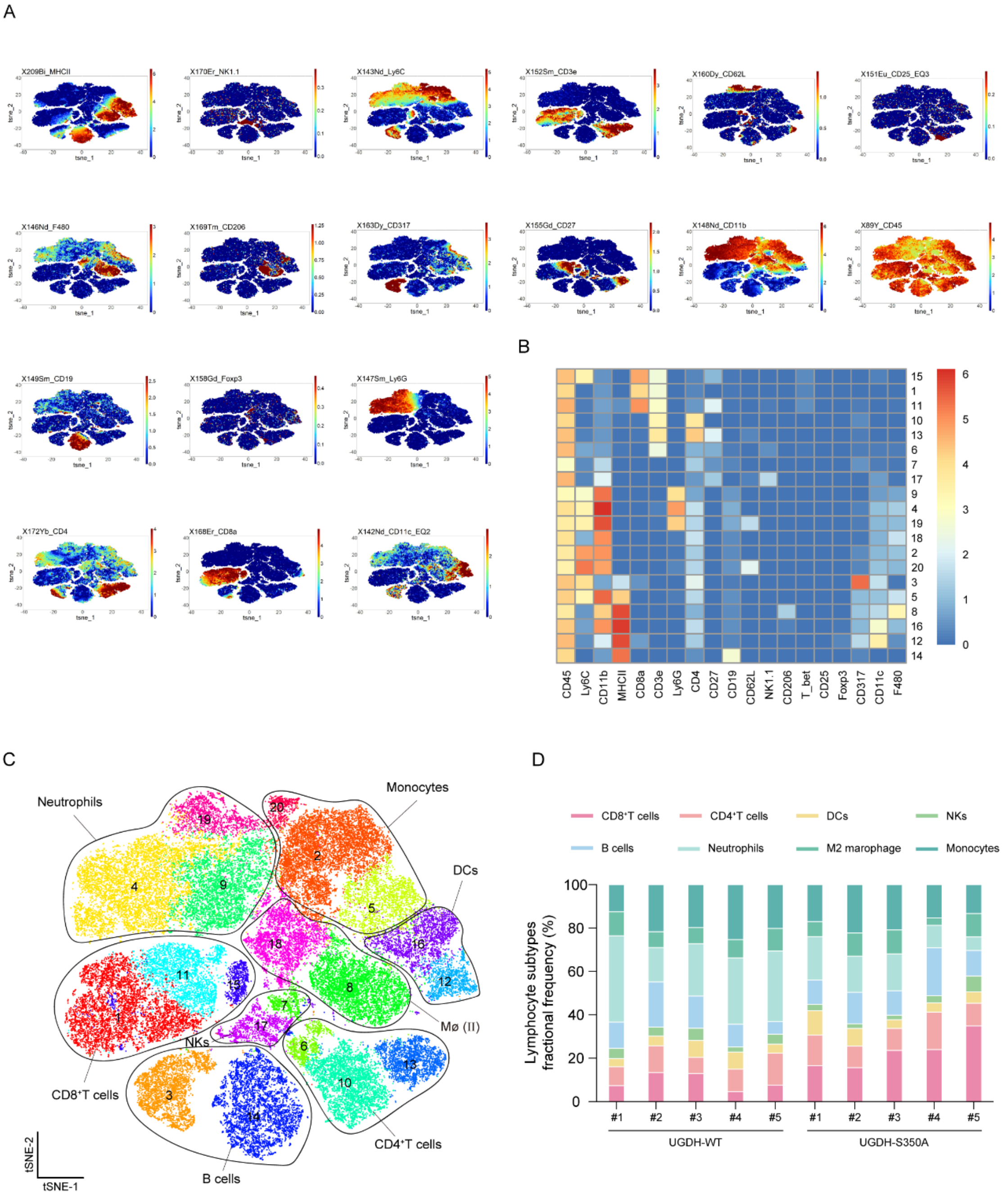

Fig. S3. Analysis of NSCLC tumor immunocyte populations with CyTOF.

(A). The t-SNE analysis of CD45^{+} cells colored by relative expression of CyTOF markers, with populations indicated.

(B). The heat map showing the normalized expression of 19 markers used for cell

clustering in 20 cell clusters. The cluster names and markers are displayed on the right and bottom sides of the heat map, respectively.

(C). The t-SNE plot graph showing the 20 cell clusters identified in total lymphocytes using the FlowSOM algorithm and overlaid with color based on cluster.

(D). Frequency plots of various lymphocyte subpopulations in various samples of WT and mutant groups.

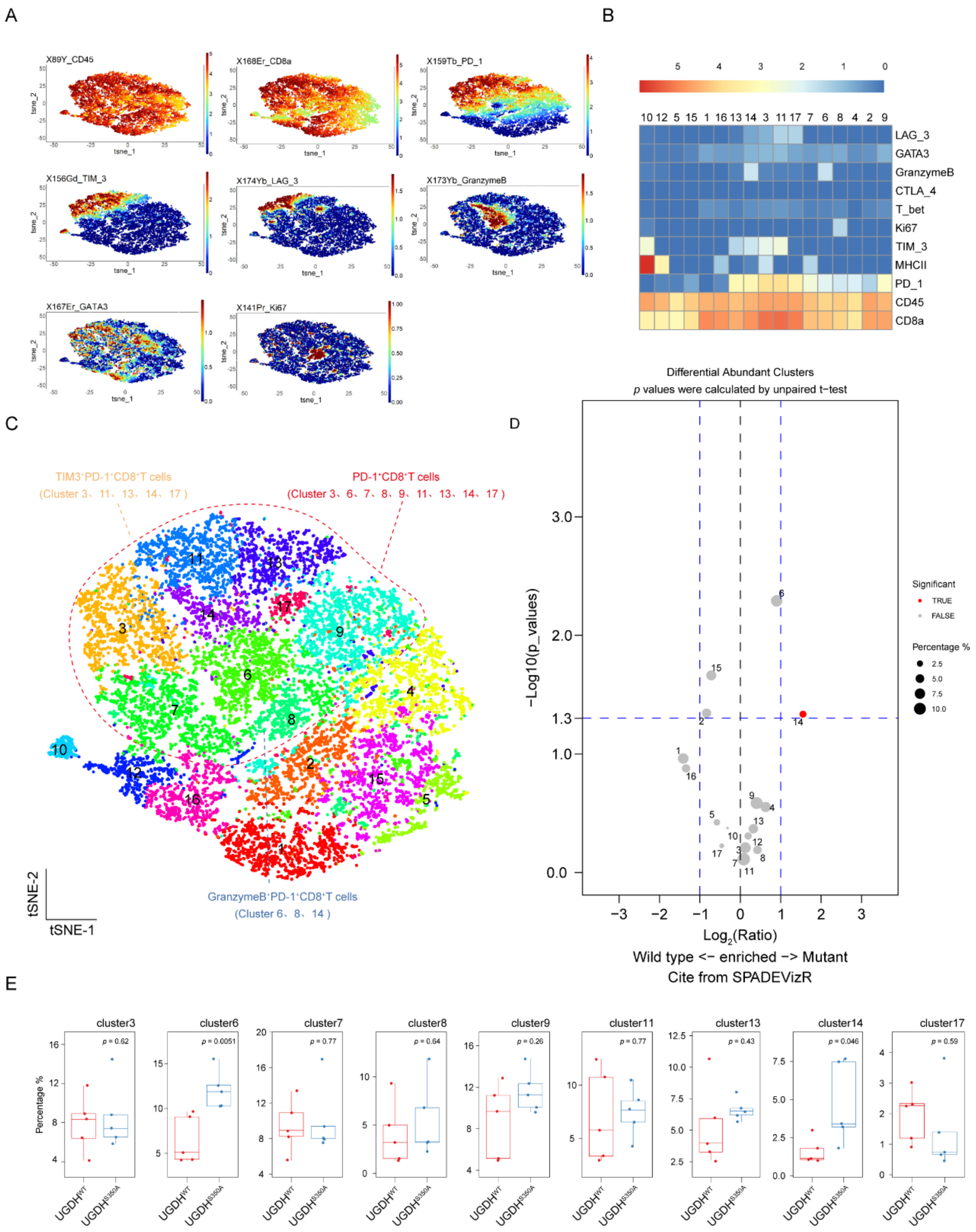

Fig. S4. The composition of CD8$^+$ T cells in tumors.

(A). The t-SNE analysis of CD8$^+$ cells colored by relative expression of CyTOF markers, with populations indicated.

(B). The heat map showing the normalized expression of 11 markers used for cell clustering in 17 CD8$^+$ T cell clusters. The cluster names and markers are displayed on the right and bottom sides of the heat map, respectively.

(C). The t-SNE plot graph showing the 17 cell clusters identified in total CD8$^+$

lymphocytes using the FlowSOM algorithm and overlaid with color based on cluster.

(D). The R package SPADEVizR method was used to analyze and display the differences in CD8$^+$ T cell clusters composition between WT and mutant groups.

(E). Comparison of the frequency of CD8$^+$ lymphocyte sub-cluster in various samples of WT and mutant groups. Data are presented as mean ± sem. with all data points indicated.

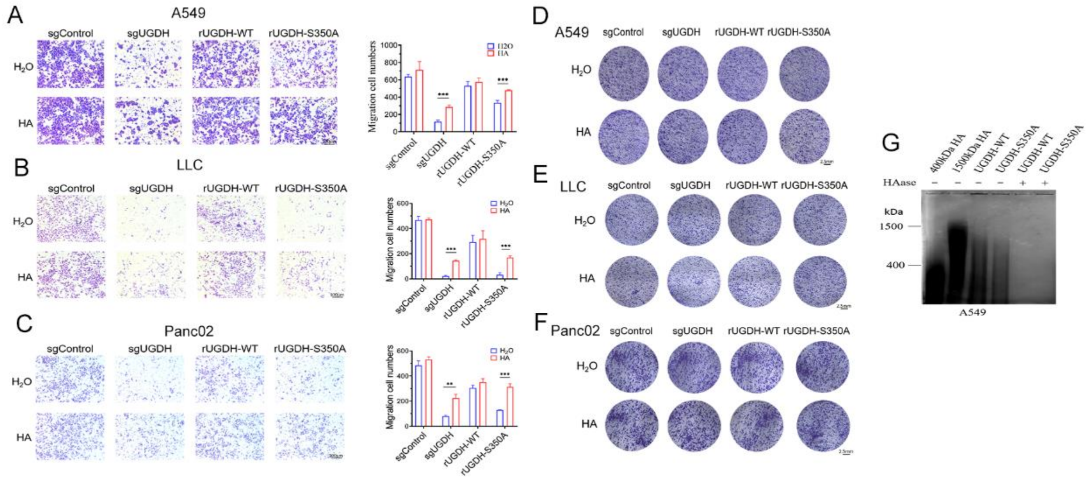

Fig. S5. The effect of HA on the migration and colonization of A549, LLC and Pan02 cells.

(A-C). The transwell assay was used to quantitatively detect the migration ability of genetically modified A549 (A), LLC (B), and Pan02 (C) cells in the presence or absence of HA (n = 3 assays).

(D-F). Representative graphs of colonization ability of engineered A549 (D), LLC (E), and Pan02 (F) cells as detected by colony formation assay in the presence or absence of HA.

(G). HA analysis by pulse-field gel electrophoresis. HAase was used to specifically degrade HA.

Data are presented as means ± SEM. Statistics by unpaired t-test.

A

Michaelis Constants for Wild-Type , S350A of UGDH in HEK293T

Parameter \ Enzyme	UDP-Glucose	
	UGDH-WT	UGDH-S350A
K_m (μM)	11.4	37.7
V_{max} (μM/min)	0.597	0.224
K_{cat} (s^{-1})	57.8	21.7
K_{cat}/K_m ($s^{-1}\cdot\mu M^{-1}$)	5.07	0.576

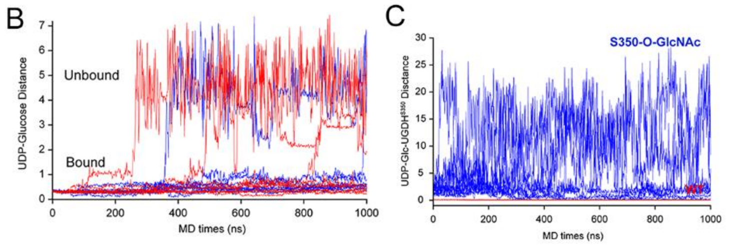

Fig. S6. The steady-state kinetics analysis of UGDH-catalyzed reactions.

(A). Michaelis constants for $UGDH^{WT}$ and $UGDH^{S350A}$. **(B).** The distance trajectory between UDP-glucose (UDP-Glc) and the binding sites derived from the MD simulations. Notably, ten simulations were conducted for each form, with red representing the WT and blue depicting the form of S350-O-GlcNAcylation form. **(C).** The contact trajectories between UDP-Glc and S350 in both WT and O-GlcNAc forms.

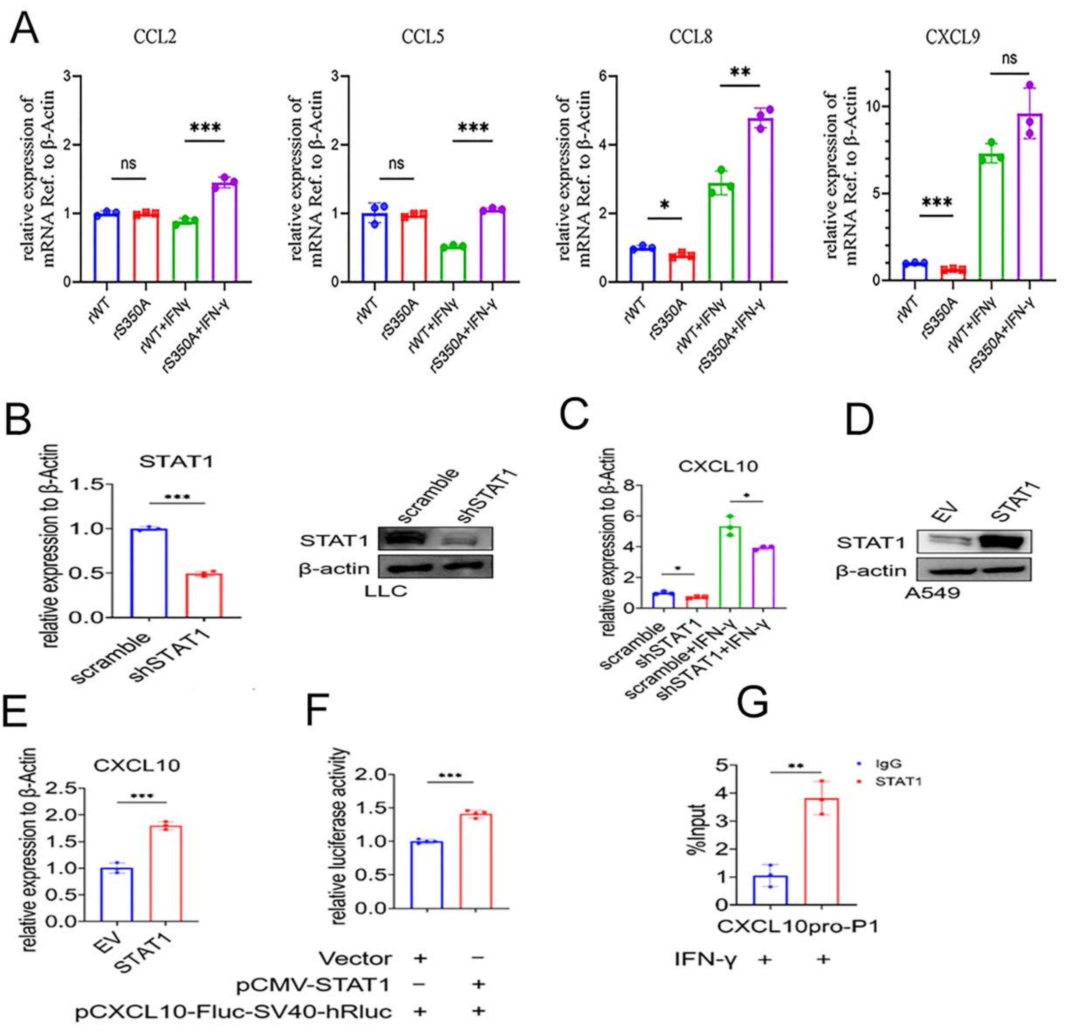

Fig. S7. STATI directly regulates CXCL10 expression in A549 cells.

(A). Quantification of CCL2, CCL5, CCL8 and CXCL9 expressions in UGDH WT and S350A cells with or without IFNγ treatment.

(B). Quantitative PCR analysis and immunoblotting analysis of STAT1 expressions in LLC cells infected with scramble or shSTAT1.

(C). The mRNA expression of CXCL10 in LLC cells infected with scramble or shSTAT1.

(D). Immunoblotting analysis of STAT1 expression in A549 cells expressing a control vector or LV-STAT1.

(E). The mRNA expression of CXCL10 in A549 cells expressing a control vector or LV-STAT1.

(F). Dual-luciferase reporter assay showing the effects of STAT1 overexpression on

relative CXCL10-promoter activity in HEK293T cells.

(G). ChIP-qPCR analysis of STAT1 binding to the regions of CXCL10 promoter in A549 cells.

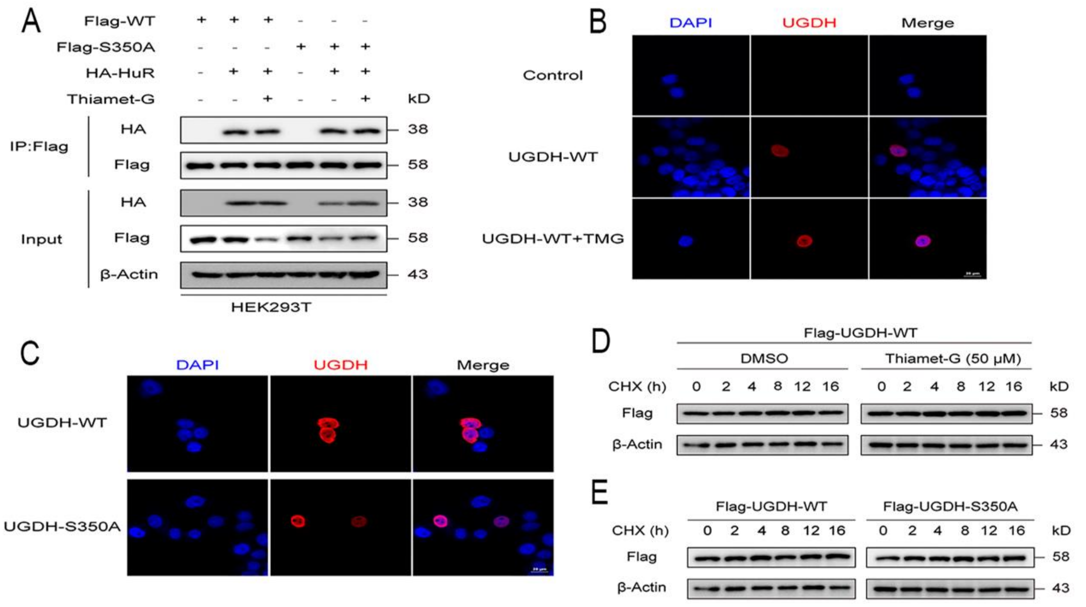

Fig. S8. O-GlcNAcylation of UGDH does not affect UGDH protein stability, subcellular localization, and interaction with HuR.

(A). Immunoblotting analysis of the interaction between UGDH and HuR in the presence or absence of TMG (50 μM).

(B). Immunofluorescence analysis of the subcellular localization of UGDH with different glycosylation levels. Scale bar, 20 μm.

(C). Immunofluorescence analysis of the subcellular localization of $UGDH^{WT}$ and $UGDH^{S350A}$. Scale bar, 20 μm.

(D-E). Immunoblotting analysis of $UGDH^{WT}$ with different glycosylation levels (D) or $UGDH^{S350A}$ (E) in Flag-UGDH expressing HEK293T cells. Cells were treated with 100 μM cycloheximide (CHX) and collected at the indicated points for analysis.

Supplementary Tables

Table S1. The cell membrane, intracellular and nuclear markers for the CyTOF panel.

No.	Antibody	Clone	Channel	Location
1	MHC Ⅱ	M5/114.15.2	209Bi	Cell membrane
2	CD62L	MEL-14	160Dy	Cell membrane
3	Inos	CXNFT	161Dy	Cytoplasm
4	CD1d	51.1	162Dy	Cell membrane
5	CD317	26F8	163Dy	Cell membrane
6	CX3CR1	2H4C9	164Dy	Cell membrane
7	T_bet	4B10	166Er	Nucleus
8	GATA3	TWAJ	167Er	Nucleus
9	CD8a	53-6.7	168Er	Cell membrane
10	NK1.1	PK136	170Er	Cell membrane
11	CD25	PC61.5	151Eu	Cell membrane
12	CD274	10F.9G2	153Eu	Cell membrane
13	CD27	O323	155Gd	Cell membrane
14	TIM-3	RMT3-23	156Gd	Cell membrane
15	Foxp3	FJK-16s	158Gd	Nucleus
16	TCRgd	UC7-13D5	165Ho	Cell membrane
17	RORγt	AFKJS-9	175Lu	Nucleus
18	CD11c	N418	142Nd	Cell membrane
19	Ly6C	HK1.4	143Nd	Cell membrane
20	KLRG1	2F1	144Nd	Cell membrane
21	CD69	H1.2F3	145Nd	Cell membrane
22	F4/80	BM8	146Nd	Cell membrane
23	CD11b	M1/70	148Nd	Cell membrane
24	CD278	C398.4A	150Nd	Cell membrane
25	Ki67	SolA15	141Pr	Nucleus
26	Ly6G	1A8	147Sm	Cell membrane
27	CD19	6D5	149Sm	Cell membrane
28	CD3e	145-2C11	152Sm	Cell membrane
29	CTLA-4	UC10-4B9	154Sm	Cell membrane
30	PD-1	29F.1A12	159Tb	Cell membrane
31	CD206	C068C2	169Tm	Cell membrane
32	CD45	30-F11	89Y	Cell membrane

33	CD44	IM7	171Yb	Cell membrane
34	CD4	RM4-5	172Yb	Cell membrane
35	GranzymeB	GB11	173Yb	Cytoplasm
36	LAG-3	C9B7W	174Yb	Cell membrane
37	Siglec-F	E50-2440	176Yb	Cell membrane
